# Study on the Influence of Chirality in the Threading of Calix[6]arene Hosts with Dialkylammonium Axles

**DOI:** 10.3390/molecules25225323

**Published:** 2020-11-15

**Authors:** Carmen Talotta, Gerardo Concilio, Paolo Della Sala, Carmine Gaeta, Christoph A. Schalley, Placido Neri

**Affiliations:** 1 Dipartimento di Chimica e Biologia “A. Zambelli”, Università di Salerno, Via Giovanni Paolo II 132, I-84084 Fisciano, Italy; ctalotta@unisa.it (C.T.); gconcilio@unisa.it (G.C.); pdellasala@unisa.it (P.D.S.); cgaeta@unisa.it (C.G.); 2 Institut für Chemie und Biochemie, Freie Universität, Arnimallee 20, 14195 Berlin, Germany; 3 School of Life Sciences, Northwestern Polytechnical University, Xi’an 710072, China

**Keywords:** calixarenes, threading, chirality, barfate salts, pseudorotaxane, chiral axles, chiral wheels.

## Abstract

The influence of chirality in calixarene threading has been studied by exploiting the “superweak anion approach”. In particular, the formation of chiral pseudo[2]rotaxanes bearing a classical stereogenic center in their axle and/or wheel components has been considered. Two kind of pseudo[2]rotaxane stereoadducts, the “*endo*-chiral” and “*exo*-chiral” ones, having the stereogenic center of a cationic axle inside or outside, respectively, the calix-cavity of a chiral calixarene were preferentially formed with specifically designed chiral axles by a fine exploitation of the so-called “*endo*-alkyl rule” and a newly defined “*endo*-α-methyl-benzyl rule” (*threading of a hexaalkoxycalix[6]arene with a directional (α-methyl-benzyl)benzylammonium axle occurs with an* endo*-α-methyl-benzyl preference*). The obtained pseudorotaxanes were studied in solution by 1D and 2D NMR, and in the gas-phase by means of the enantiomer-labeled (EL) mass spectrometry method, by combining enantiopure hosts with pseudoracemates of one deuterated and one unlabeled chiral axle enantiomer. In both instances, there was not a clear enantiodiscrimination in the threading process with the studied host/guest systems. Possible rationales are given to explain the scarce reciprocal influence between the guest and host chiral centers.

## 1. Introduction

Over the past two decades, there has been a great scientific interest for the synthesis of mechanomolecules [1], such as rotaxanes and catenanes [2,3,4]. Mechanomolecules have found many applications in very different topics, such as nanoelectronics [5,6,7], molecular machines [2,3,8,9,10], and catalysis [11,12,13]. The mechanical bond [1], as a building element of rotaxane and catenane architectures, is usually obtained by template-directed synthesis [14] based on the threading of a linear molecule (axle) through a macrocyclic component (wheel). The most convenient synthetic routes are the threading-followed-by-stoppering, the clipping, and the slipping methods [1,14] that exploit weak intermolecular interactions (hydrogen bond, halogen bond, π-stacking, cation-π, or metal coordination) to assemble the molecular components. 

**Figure 1:**
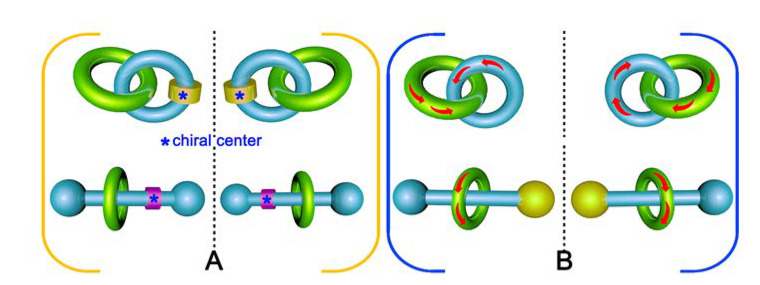
Chiral catenanes and rotaxanes. Chirality arising from (**A**) classical chiral elements and (**B**) “mechanical chirality” with achiral “directional” elements.

The peculiar stereochemical features of mechanomolecules are one of the most fascinating aspects of these architectures. In particular, the chirality within such supramolecular architectures [15,16] is of special interest, because it is relevant for enantioselective recognition and sensing [17], asymmetric catalysis [13,18,19,20], and unidirectional molecular motors [21,22,23]. The simplest way to obtain chiral rotaxanes and catenanes is to introduce a classical stereogenic element in one their components to give a chiral axle and/or a chiral wheel (Figure 1A) [16,24,25]. Another more sophisticated approach is given by “mechanical chirality” [26,27,28,29,30] (Figure 1B) in which chirality arises from the combination of achiral “directional” elements spatially restricted by the mechanical bond. Of course, the first approach is more convenient from the synthetic point of view, because a difficult resolution step, on an appropriate preparation scale, can be avoided by resorting to suitable enantiopure moieties available from the chiral pool. 

**Scheme 1:**
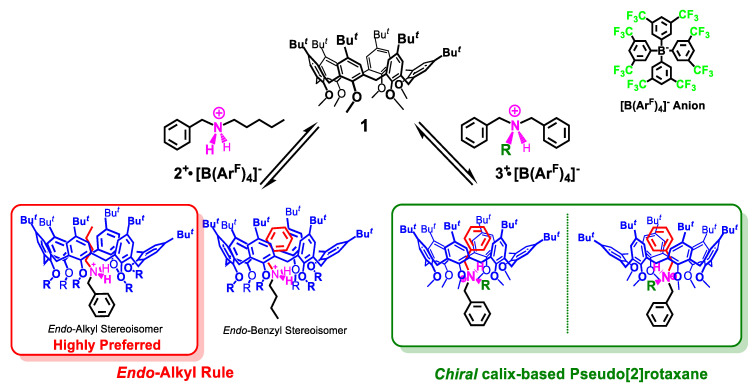
Structures of calix[6]arene host **1**, alkylammonium axles **2^+^** and **3^+^**, BArF anion, examples of classical and chiral pseudo [2] rotaxane.

Over the past ten years, our group has shown that the threading of scarcely preorganized calix[6]arene macrocycles (e.g., **1**, Scheme 1) with dialkylammonium axles (e.g., **2^+^**) occurs in CDCl_3_ when the ammonium linear system is coupled with the weakly coordinating barfate anion tetrakis[3,5-bis(tri-fluoromethyl)phenyl]borate [B(Ar^F^)_4_]^−^ “superweak anion” [31,32,33,34,35]. This method was defined, in short, as the “superweak anion approach” [31,32,33,34]. Interestingly, we also observed that the threading of alkylbenzylammonium cations may result in two different stereoisomeric pseudorotaxanes [32], in which the alkyl or the benzyl moiety is hosted inside the calixarene cavity. These have been termed as “*endo*-alkyl” or “*endo*-benzyl” isomers, respectively (Scheme 1). The *endo*-alkyl “orientational mechanostereoisomer” is usually preferred [32], thus leading to the definition of the so-called “*endo*-alkyl rule”: *threading of a directional alkylbenzylammonium axle through a hexaalkoxycalix[6]arene occurs with an* endo*-alkyl preference* [36].

A full exploitation of the “*endo*-alkyl rule” within the “superweak anion approach” has led to several interesting examples of stereoprogrammed calixarene-based pseudorotaxanes, to their integrative self-sorting [36], and to the synthesis of the corresponding rotaxane and catenane mechanomolecules [37,38].

The threading of a tertiary ammonium axle [39] has led to the first examples of dissymmetric calixarene-based pseudo[2]rotaxanes (Scheme 1, lower right) obtained by combination of two achiral components. In detail, this peculiar chirality was generated by the structural directionality of calix[6]arene macrocycle which led to differentiate the two benzyl units of the prochiral tertiary ammonium axle [39]. Interestingly, this represents an example of a dissymmetric pseudorotaxane in which an atomic stereogenic center is generated by the threading of an axle with a directional wheel. 

**Chart 1:**
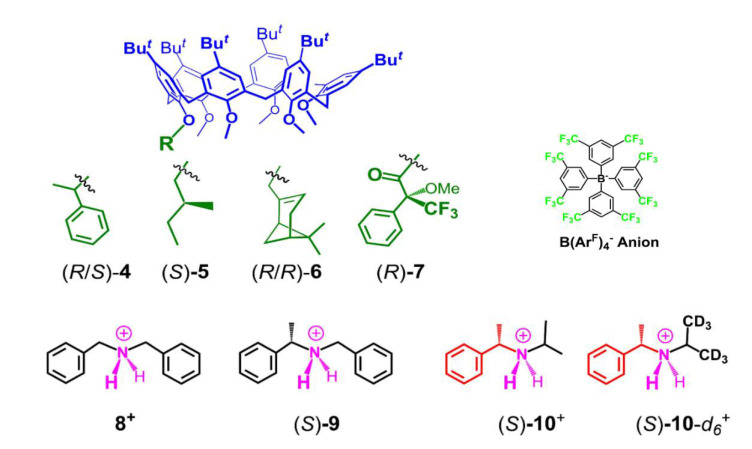
Structures of chiral calix[6]arene hosts **4–7**, alkylammonium axles **8^+^**, **9^+^**, **10^+^**, **10-***d_6_***^+^**, and [B(Ar^F^)_4_]^−^ anion.

On the basis of the above background and considering the great potential of chiral calixarene-based MIMs in different fields such as enantioselective recognition, sensing, and catalysis, we decided to synthesize chiral calix-wheels by introducing chiral pendant groups on the calix[6]arene scaffold (**4**–**7**, Chart 1) and to study their threading with chiral secondary dialkylammonium cations (**9^+^**–**10^+^**) by exploiting the superweak anion approach. 

## 2. Results and Discussion 

### 2.1. Synthesis of Chiral Calixarenes

**Scheme 2:**
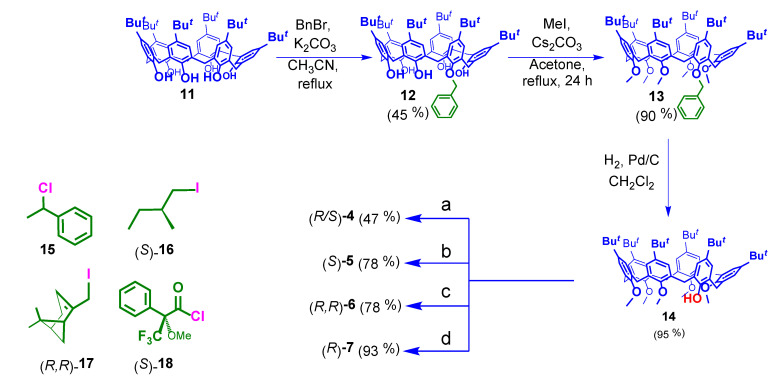
Synthesis of chiral calixarene derivatives **4**–**7**. (**a**) **15**, NaH, *dry*-DMF 80 °C, 12 h. (**b**) **16**, NaH, *dry*-DMF, 80 °C, 12 h. (**c**) **17**, NaH, *dry*-DMF, 80 °C, 12 h. (**d**) **18**, DMAP, *dry*-NEt_3_, *dry*-DMF, 70 °C, 12 h.

Pentamethoxy-calix[6]arene-mono-ol **14** [40,41,42] was the ideal precursor for the synthesis of chiral calixarene derivatives (*R/S*)-**4**, (*S*)-**5**, (*R*)-**6**, and (*R*)-**7** (Scheme 2) obtainable by the introduction (alkylation or esterification) of a chiral pendant moiety on its hydroxyl group located at the lower rim.

Derivative **14** was synthesized by following the reaction sequence reported in Scheme 2 [40,41,42]. In particular, native *p-tert*-butylcalix[6]arene **11** was monobenzylated using benzyl bromide and K_2_CO_3_ to give calix[6]arene **12** in 45% yield [42]. Derivative **12** was exhaustively methylated by treatment with MeI in acetone using Cs_2_CO_3_ as a base to give calix[6]arene **13** in 90% yield [42]. The removal of the benzyl group was easily accomplished by hydrogenolysis to give mono-ol **14** [42]. 

The treatment of **14** with α-methylbenzylbromide in the presence of NaH leads to the racemic compound (*R/S*)-**4** in 47% yield. 

**Figure 2:**
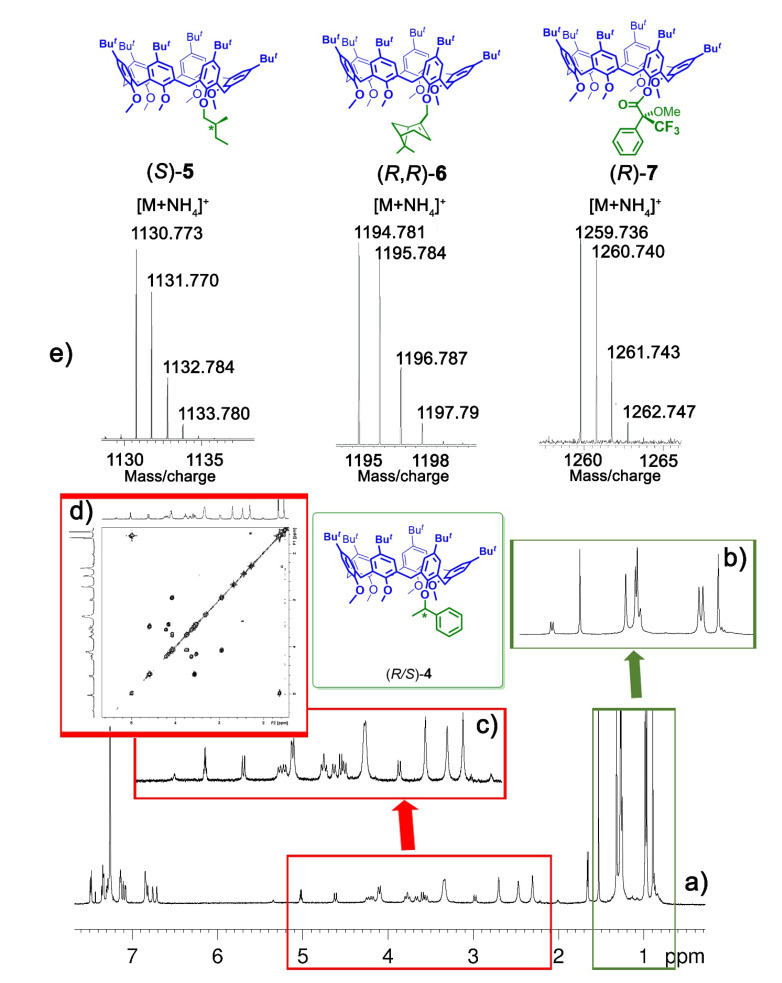
^1^H NMR spectrum (CDCl_3_, 600 MHz) of derivative (*R/S*)-**4** at 298 K (**a**). Different portions of its ^1^H NMR (**b**,**c**) and COSY−45 (**d**) spectrum. Partial ESI mass spectra of compounds (*S*)-**5**, (*R*,*R*)-**6**, and (*R*)-**7** (**e**).

The ^1^H NMR spectrum (400 MHz, CDCl_3_) of (*R/S*)-**4** at 298 K (Figure 2b) shows six *^t^*Bu singlets at 0.89, 0.96, 0.98, 1.27, 1.28, and 1.32 ppm (1:1:1:1:1:1 ratio) while the ArCH_2_Ar groups gave rise to six sharp AX systems at 4.62/3.59 (*J* = 13.4 Hz), 4.18/3.55 (*J* = 14.0 Hz), 4.24/3.66 (*J* = 14.0 Hz), 4.11/3.75 (*J* = 13.6 Hz), 4.09/3.79 (*J* = 13.9 Hz), 4.10/2.97 (*J* = 13.9 Hz). The C*H*(CH_3_) group shows a resonance at 5.02 ppm (quadruplet, *J* = 6.1 Hz, 1H), while the methyl group resonates at 1.66 ppm as a doublet (*J* = 6.1 Hz). In addition, aromatic signals attributable to the benzyl group at the lower rim resonate at 6.75–7.53 ppm. 

Chiral calixarenes in enantiopure form (*S*)-**5**, (*R,R*)-**6**, and (*R*)-**7** (Scheme 2) were obtained by a similar functionalization of the OH group of calix[6]arene-mono-ol **14** with enantiopure chiral reagents. All four compounds were fully characterized by NMR and MS (Figure 2e) spectroscopy (see the experimental section and SI).

### 2.2. Syntheses of Chiral Axles

**Scheme 3:**
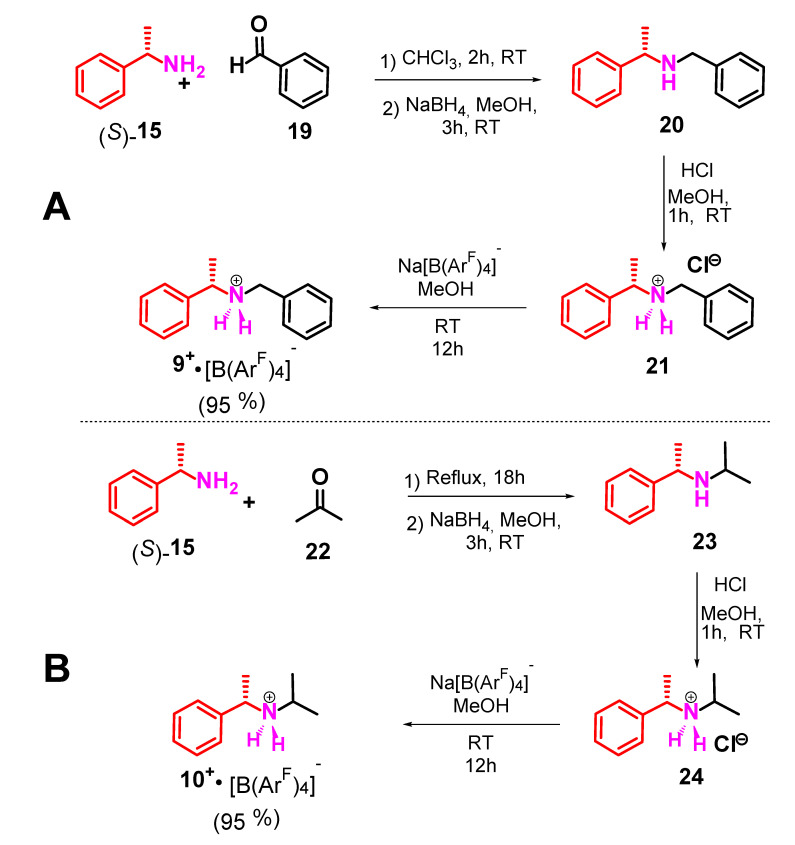
Synthesis of enantiopure derivatives (*S*)-**9^+^**∙[B(Ar^F^)4]^−^ (**A**) and (*S*)-**10^+^**∙[B(Ar^F^)4]^−^ (**B**).

With the aim to observe an enantiodiscrimination effect in the formation of pseudo[2]rotaxane, the new linear chiral derivatives (*S*)-**9^+^**∙[B(Ar^F^)_4_]^−^, (*S*)-**10^+^**∙[B(Ar^F^)_4_]^−^, and (*S*)-**10-***d_6_***^+^**∙[B(Ar^F^)_4_]^−^ were designed. The synthesis of (*S*)-**9^+^**∙[B(Ar^F^)_4_]^−^ is outlined in Scheme 3A. A mixture of (*S*)-α-methyl-benzylamine (*S*)-**15** and benzaldehyde **19** in chloroform was stirred for 2 h at room temperature and then directly reduced with NaBH_4_. The secondary amine was treated with HCl/MeOH to give chloride salt **21**. Finally, a counterion exchange with NaBAr^F^ led to (*S*)-**9^+^**∙[B(Ar^F^)_4_]^−^ salt. In a similar way, (*S*)-**10^+^**∙[B(Ar^F^)_4_]^−^ was synthesized from (*S*)-α-methyl-benzylamine (*S*)-**15** and acetone **22** at reflux for 18 h and then reduced with NaBH_4_. HCl (37%) was then used to obtain the chloride salt which was exchanged with Na[B(Ar^F^)_4_]^−^ (Scheme 3B).

**Scheme 4:**
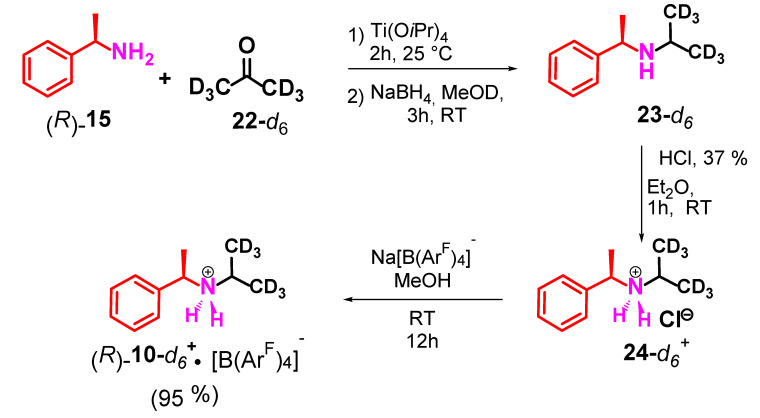
Synthesis of enantiopure derivative (*R*)-**10**-*d_6_***^+^**∙[B(Ar^F^)4]^−^.

The synthesis of (*S*)-**10**-*d_6_***^+^**∙B(Ar^F^)_4_^−^ (Scheme 4) was obtained from the amine-acetone coupling, by using Ti(O*i*Pr)_4_ as a catalyst (see experimental section for further details). The acidification with HCl (37%) gave the ammonium chloride and the usual salt exchange gave the required axle. In a similar way, the corresponding (*R*)-axles were also made in order to have access to the pseudoracemates. 

### 2.3. NMR Threading Studies of Chiral Calixarene (R/S)-***4***

#### 2.3.1. Threading of Racemic Calix[6]arene-wheel (*R/S*)-**4** with Dibenzylammonium Axles **8^+^** and **9^+^**

**Scheme 5:**
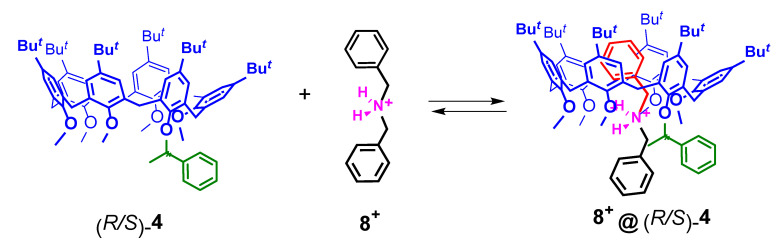
Formation of the **8^+^@**(*R/S*)-**4** pseudo[2]rotaxanes.

*Through-the-annulus* threading with linear axles was initially studied by using NMR spectroscopy. As an initial study, we decided to investigate the complexing ability of racemic (*R/S*)-**4** towards achiral dibenzylammonium cation **8^+^** (Scheme 5).

**Figure 3:**
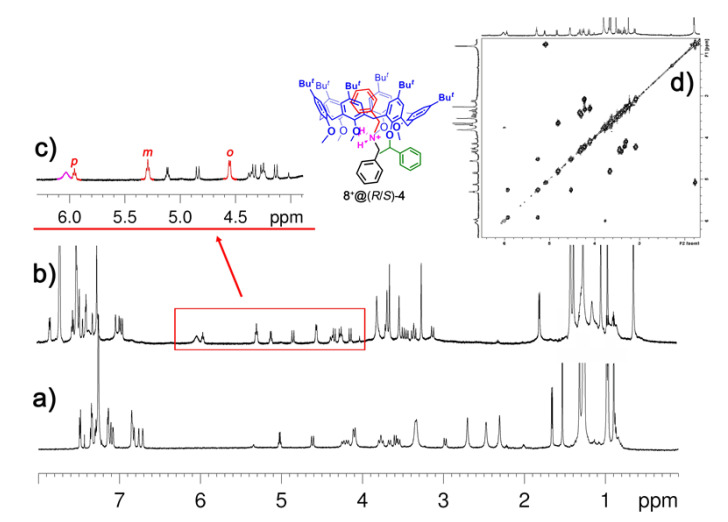
Significant portions of the ^1^H NMR spectra (600 MHz, CDCl_3_, 298 K) of: (**a**) (*R/S*)-**4**; (**b**) 1:1 mixture (3 mM) of (*R/S*)-**4** and **8^+^**∙B(Ar^F^)_4_^−^ after mixing. Insets: (**c**) expansion of the ^1^H NMR and (**d**) COSY portions of the mixture; the BnH protons of the shielded *endo*-cavity benzylammonium moiety are marked in red.

The ^1^H NMR spectrum of a 1:1 mixture of (*R/S*)-**4** and **8^+^**∙B(Ar^F^)_4_^−^ salt in CDCl_3_ at 25 °C (Figure 3) showed, immediately after mixing, important changes that confirmed the formation of the pseudorotaxane [32] **8^+^@**(*R/S*)-**4** (Scheme 5) by a threading equilibrium slow on the NMR timescale. In details, shielded aromatic signals attributable to the benzylic unit (*ortho*-BnH, *meta*-BnH, and *para*-BnH) hosted inside the calixarene cavity, were detected respectively at 4.55, 5.29, and 5.95 ppm, while the benzylic-CH_2_ was found at 5.11 ppm (Figure 3b,c) [32].

The spectrum remained unchanged after 12 h at 55 °C, thus demonstrating that the system had reached the equilibrium immediately after the mixing. A 2D COSY spectrum (Figure 3d) allowed the assignment of all the relevant resonances of **8^+^**@(*R/S*)-**4** racemic pseudo[2]rotaxane.

**Figure 4:**
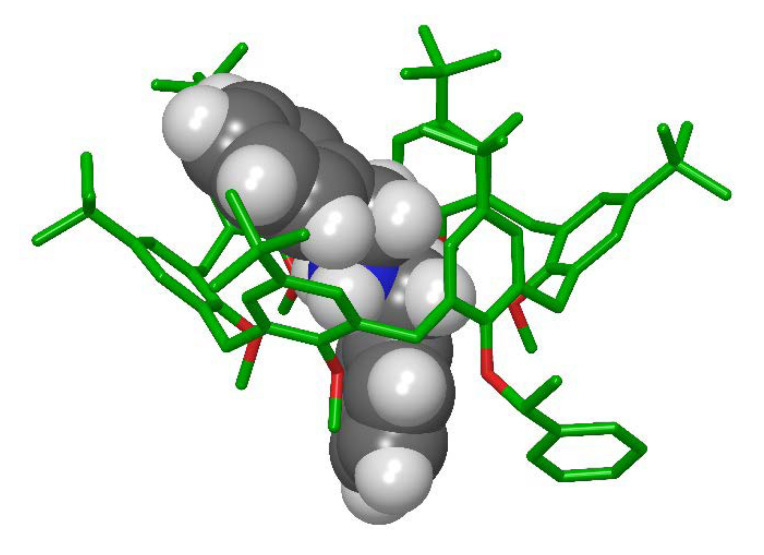
DFT-optimized structures of **8^+^@**(*R*)-**4** pseudo[2]rotaxane at the B97D3/SVP/SVPFIT level of theory.

DFT calculations at the B97D3/SVP/SVPFIT level of theory were performed on pseudorotaxane **8^+^**@(*R*)-**4**. A close inspection of the optimized pseudo[2]rotaxane structure (Figure 4) reveals the presence of typical H-bonds between the ammonium group of the dibenzylic **8^+^** axle and the oxygen atoms at the calix[6]arene lower rim (average: N···O distance of 3.6 Å and N-H···O angle of 158.7°). In addition, C-H···π interactions between the H-atoms of the axle and the calixarene aromatic walls, (C-H···π*^centroid^* distance of 2.9 Å and C-H···π*^centroid^* angle of 131.1°) [43] play a crucial role in the stabilization of the supramolecular structure. 

**Scheme 6:**
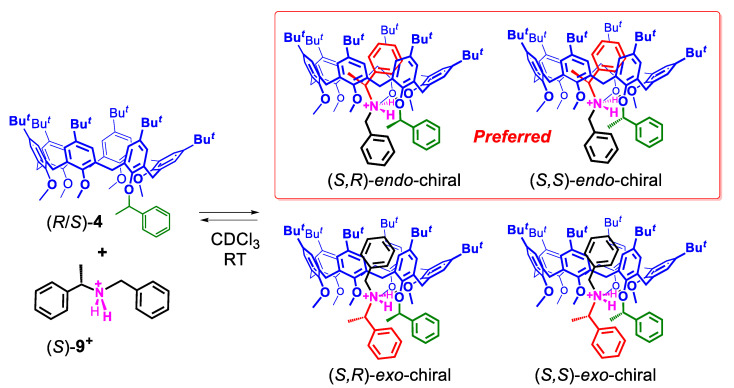
Stereoisomeric pseudo[2]rotaxanes obtainable by threading of racemic (*R/S*)-**4** with enantiopure axle (*S*)-**9^+^**.

**Figure 5:**
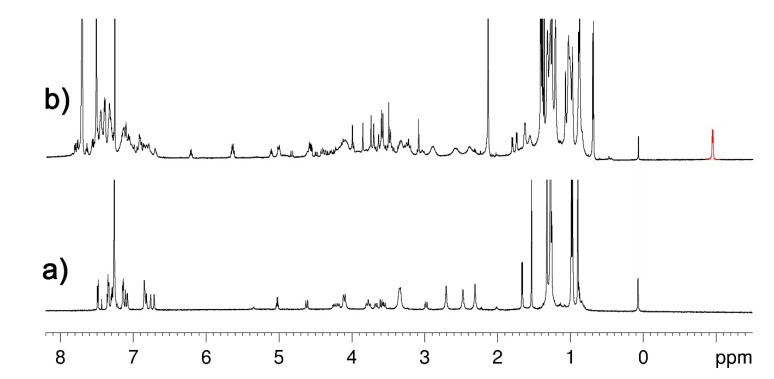
^1^H NMR spectra (600 MHz, CDCl_3_, 298 K) of: (**a**) (*R/S*)-**4**. (**b**) 1:1 mixture (3 mM) of (*R/S*)-**4** and (*S*)-**9^+^**∙B(Ar^F^)_4_^−^. Marked in red the doublet attributable to the methyl group of the axle shielded inside the calixarene cavity.

**Figure 6:**
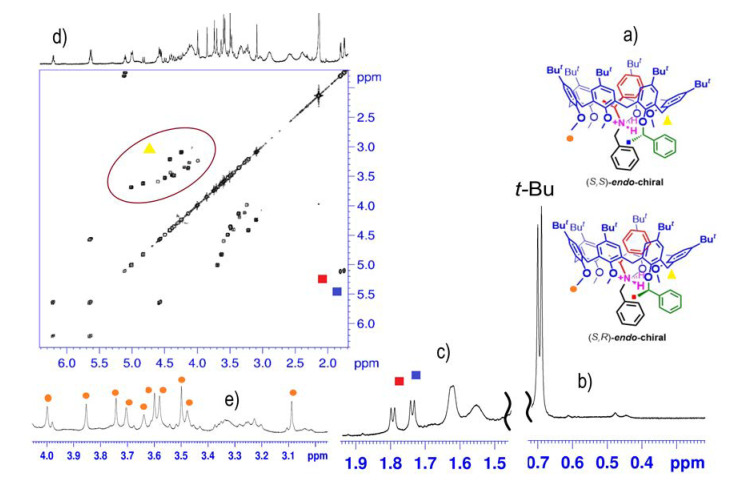
(**a**) Chemical drawing of the two possible *endo*-chiral (*S*)-**9^+^@**(*R*)-**4** and (*S*)-**9^+^@**(*S*)-**4** pseudorotaxanes. (**b**,**c**,**e**) Expansions of the ^1^H NMR spectrum (600 MHz, CDCl_3_, 298 K) showing the signals of the two (*S*)-**9^+^@**(*R*)-**4** and (*S*)-**9^+^@**(*S*)-**4** diastereoisomers, in equal ratio. (**d**) Expansion of the COSY spectrum of the 1:1 mixture (3 mM) of (*R/S*)-**4** and (*S*)-**9^+^**∙B(Ar^F^)_4_^−^, marked the 12 ^2^*J*-correlations attributable to the methylene groups of the two stereoisomers.

With these results in hand we turned our attention to the study of the threading of calixarene (*R/S*)-**4** with the enantiopure dibenzylammonium axle (*S*)-**9^+^**∙B(Ar^F^)_4_^−^ (Scheme 6). In this specific case, the formation of a pseudo[2]rotaxane could lead to four different stereoisomers: two “*endo*-chiral” stereoadducts with the stereogenic center of cation **9^+^** inside the calix-cavity for both calixarene enantiomers (being the calixarene in racemic form) and two “*exo*-chiral” ones with the stereogenic center outside the cavity (Scheme 6). The ^1^H NMR spectrum of an equimolar (3 mM) solution of (*R/S*)-**4** and (*S*)-**9^+^**∙B(Ar^F^)_4_^−^ (Figure 5 and Figure 6) showed the formation of only “*endo*-chiral” stereoisomers. In fact, we can observe a diagnostic doublet at negative values (in red in Figure 5b) attributable to the methyl group of the α-methylbenzyl moiety shielded by the aromatic walls.

In addition, shielded *endo*-cavity benzyl resonances were present in the 4.8–6.5 ppm range (Figure 5b). A 2D-COSY spectrum (Figure 6d and Supplementary Materials) showed all the basic correlations necessary to confirm the formation of the “*endo*-chiral” (*S*)-**9^+^@**(*R*)-**4** and (*S*)-**9^+^@**(*S*)-**4** pseudo[2]rotaxane diastereoisomers. 

Note that this result represents an important extension of the above mentioned “*endo*-alkyl rule”, because it demonstrates that the calix-cavity prefers to host the α-methyl-benzyl moiety with respect to the simple unsubstituted benzyl group. Evidently, the α-methyl group gives rise to additional stabilizing interactions with the aromatic walls which direct the preferential formation of the “*endo*-chiral” diastereoisomers - an information of pivotal importance in the design of new calixarene-based MIMs.

On this basis, a new stereoselectivity rule (named as “*endo*-α-methyl-benzyl rule”) for the threading of calixarene macrocycles was defined: *threading of* *a hexaalkoxycalix[6]arene with a directional (α-methyl-benzyl)benzylammonium axle occurs with an* endo*-α-methyl-benzyl* preference. 

DFT calculations (vide infra) confirmed that the α-methyl group gives rise to additional stabilizing C‒H···π interactions with the calixarene aromatic walls which leads to the preferential formation of the “*endo*-chiral” diastereoisomer. 

**Figure 7:**
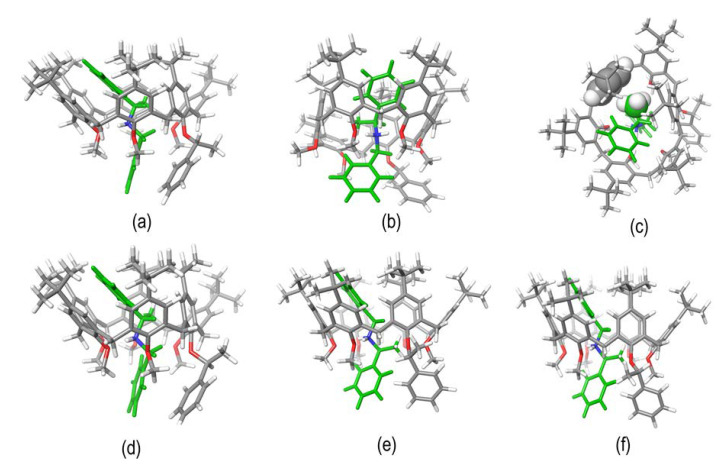
DFT optimized structures at the B97D3/SVP/SVPFIT level of theory of the stereoisomeric *exo-*chiral and *endo*-chiral **9^+^@4** pseudorotaxanes. (**a**) *endo*-chiral (*S*)-**9^+^@**(*S*)-**4** pseudorotaxane. (**b**) Particular of the ^+^NH*^axle^*···OR*^calix^* H-bonding interactions in (*S*)-**9^+^@**(*S*)-**4**. (**c**) Particular of the C-H···π interactions in (*S*)-**9^+^@**(*S*)-**4**. (**d**) *endo*-chiral (*S*)-**9^+^@**(*R*)-**4** pseudorotaxane. (**e**) *exo*-chiral (*S*)-**9^+^@**(*R*)-**4** pseudorotaxane. (**f**) *exo*-chiral (*S*)-**9^+^@**(*S*)-**4** pseudorotaxane.

Furthermore, a quantitative evaluation about the number and integrals of the signals (Figure 6) in the methoxy (Figure 6e) and *tert*-butyl (Figure 6b) regions of the spectrum, indicated that the two (*S*)-**9^+^@**(*R*)-**4** and (*S*)-**9^+^@**(*S*)-**4** pseudo[2]rotaxane diasteroisomers were formed in equal ratio. This means that no enantiodiscrimination was observed in their formation probably because the two chiral centers were too remote from each other to exert a mutual influence. In accord with this experimental observation, DFT calculations at the B97D3/SVP/SVPFIT level of theory indicated a very slight energy difference between the two (*S*)-**9^+^@**(*R*)-**4** and (*S*)-**9^+^@**(*S*)-**4** *endo*-chiral pseudorotaxane stereoisomers (Figure 7). 

Single point energy DFT calculations (Figure 7), evidenced a significant energy difference between the *endo*-chiral and *exo*-chiral stereoisomers in Scheme 6 (ΔE = E_exo-chiral_ − E_endo-chiral_ > 3.0 kcal/mol), that corroborated the experimental results. A close inspection of the DFT optimized structure of (*S*)-**9^+^@**(*R*)-**4** *endo*-chiral pseudorotaxane (Figure 7a,b) evidenced the presence of two classical ^+^NH*^axle^*···OR*^calix^* H-bonding interactions with a N···O mean distance of 2.85 Å and a N-H···O mean angle of 160° (SI). In addition, stabilizing C-H···π interactions (Figure 7c and SI) were found for the (*S*)-**9^+^@**(*S*)-**4** *endo*-chiral pseudo[2]rotaxane between the α-methyl group of the axle of (*S*)-**9^+^** and the calixarene aromatic walls with a C-H···π*^centroid^*distance of 2.49 Å (SI). 

The DFT optimized structure of (*S*)-**9^+^@**(*R*)-**4** *exo*-chiral pseudorotaxane, showed that the α-methyl group of axle (*S*)-**9^+^** lies on the same plane of the oxygen-calixarene atoms (Figure 7e). Thus, because of the steric hindrance between the methyl group of the axle and the calixarene OR groups, a slight distortion of the geometrical H-bonding parameters was found. In particular, the ^+^NH*^axle^*···OR*^calix^* interactions showed a longer N···O mean distance of 3.02 Å and a smaller N-H···O mean angle of 145° (SI). These values are indicative of weaker H-bonding interactions between the axle (*S*)-**9^+^** and the wheel (*R*)-**4** in (*S*)-**9^+^@**(*R*)-**4** *exo*-chiral pseudorotaxane. 

#### 2.3.2. Threading of Racemic Calix[6]arene-wheel (*R/S*)-**4** with Isopropylbenzylammonium Axle (*S*)-**10^+^**

We envisioned that to improve the enantiodiscrimination, the distance between the two chiral centers should be reduced. This can be done by exploiting the “*endo-*alkyl rule” with an alkylbenzylammonium axle bearing the chiral center on the benzyl side. For this reason, we designed and synthesized derivative (*S*)-**10^+^**∙[B(Ar^F^)_4_]^−^ (see above).

**Scheme 7:**
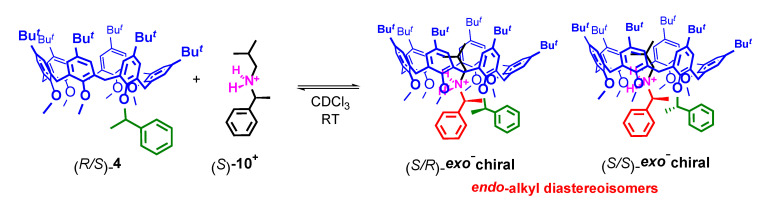
Formation of the two “*exo*-chiral” (*S*)-**10^+^@**(*R/S*)-**4** pseudo[2]rotaxane diastereoisomers.

Again, the combination of (*S*)-**10^+^**∙[B(Ar^F^)_4_]^−^ and racemic wheel (*R/S*)-**4** may (Scheme 7) could result in the formation of four stereoisomers: two “*endo*-chiral” and two “*exo*-chiral” stereoadducts with the isopropyl moiety outside or inside the calix cavity, respectively.

**Figure 8:**
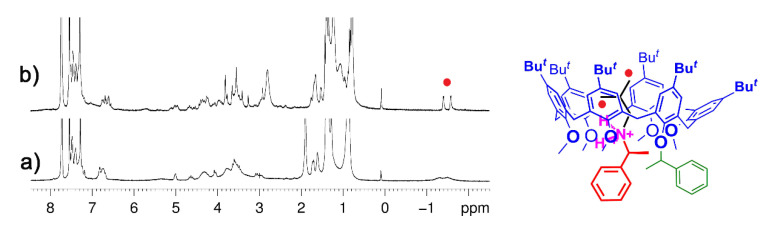
^1^H NMR spectra (CDCl_3_, 400 MHz, 298 K) of equimolar solution (3 mM) of (*R/S*)-**4** and (*S*)-**10^+^**∙[B(Ar^F^)_4_]^−^ at: (**a**) 298 K and (**b**) 243 K.

The ^1^H NMR spectrum of a 1:1 mixture (3 mM) of (*R/S*)-**4** with (*S*)-**10^+^**∙[B(Ar^F^)_4_]^−^ salt in CDCl_3_ at 25 °C (Figure 8) showed, immediately after mixing, the sharpening of all signals and the peculiar presence two doublets for the diastereotopic methyl protons of the *endo*-isopropyl group. This means that the “*exo*-chiral” orientation is preferred leading to (*S*)-**10^+^@**(*R/S*)-**4** pseudo[2]rotaxane diastereoisomers in accordance with the *endo*-alkyl rule.

In order to establish if any enantiodiscrimination is occurring in the formation of (*S*)-**10^+^@**(*R/S*)-**4** stereo-adducts we carefully examined different regions of its ^1^H NMR spectrum, but no conclusive results were obtained because of the large number of partially overlapping signals. 2D NMR techniques were also used with no conclusive results.

### 2.4. MS Experiments of the Threading of Chiral Calixarene (S)-***5***, (R)-***6***, and (R>)-***7***

To obtain more straightforward insight, enantiomer-labeled (EL) mass spectrometry was used. A fundamental feature of this method is the use of a pseudoracemate, i.e., a 1:1 mixture of one deuterated and the opposite, non-labeled enantiomer. The two possible diastereomeric pseudorotaxanes would then have different masses and are, thus, distinguishable. A 1/1 mixture of labelled and unlabelled guest enantiomers was mixed with 0.5 equivalent of the target chiral host. It was considered the competitive equilibrium system through Equations (1) and (2).

(1) $$H+G_{S}^{+}\;\begin{array}{c} K_{S}\\ ⥨ \end{array}{\left(HG_{S}\right)}^{+}$$

(2) $$H+G_{R}^{+}\;\begin{array}{c} K_{R}\\ ⥨ \end{array}{\left(HG_{R-d6}\right)}^{+}$$

Therefore, the peak intensity ratio, I_S_/I_R-_*_d6_ =* I[(HG_S_)^+^]/I[(HG_R-*d6*_)^+^], of the diastereomeric host-guest complex ions, was expected to become a measure of the enantio-discrimination ability of the host toward the two enantiomers of the chiral guest.

- **I_S_/I_R-_*_d6_*> 1** means that a given chiral host binds more strongly the (*S*)-enantiomer (the larger the I_S_/I_R-_*_d6_*ratio value, and higher the degree of chiral discrimination by the host).
- **I_S_/I_R-_*_d6_ <* 1** means that a given chiral host binds more strongly the (*R*)-labelled guest.
- **I_S_/I_R-_*_d6_*= 1.0 ± 0.1** means that a given chiral host cannot differentiate the chirality of a given guest.

#### 2.4.1. Concentration Effect

The solution concentration can affect the ionization process and, hence, the intensity ratio I[(HG_S_)^+^]/I[(HG_R-*d6*_)^+^]. Therefore, the first step was to choose the best concentration value to use. It was seen that the peak intensities were quite low up to a 300 μM solution and the intensity ratio did not change increasing the concentration over 300 μM. Therefore, 300 μM solutions were used in determining isotopic and chiral recognition effects.

#### 2.4.2. Isotopic Effects

**Figure 9:**
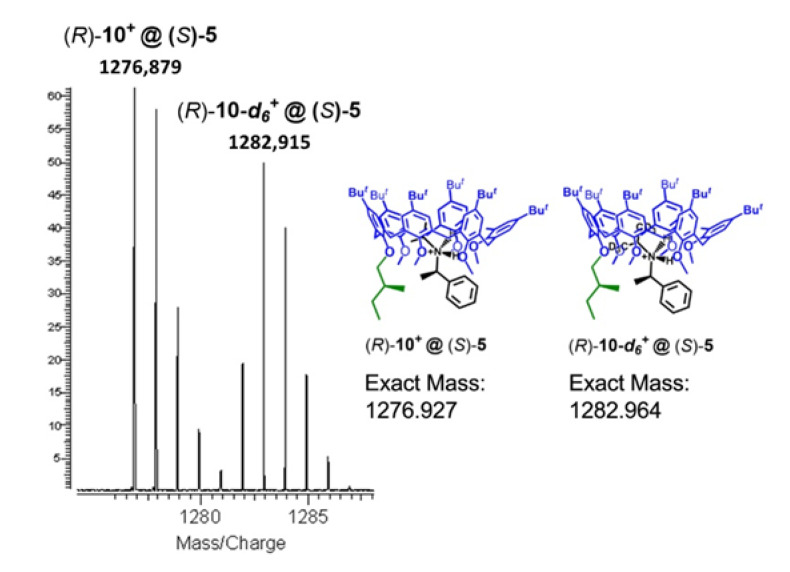
Significant portion of the mass spectrum of a 1:2:2 mixture (CH_2_Cl_2_, 300 μM) of derivatives (*S*)-**5**, (*R*)-**10^+^**·[B(Ar^F^)_4_]^−^, and (*R*)-**10**-*d_6_***^+^**·[B(Ar^F^)_4_]^−^, respectively.

Of course, isotope effects may also operate in addition to any stereochemical effect. However, this can easily be tested by a control experiment with either the oppositely labeled pseudoracemate of axles or by repeating the same experiment with the other enantiomer of the calixarene. To test, whether isotope effects play a significant role, a control experiment in Figure 9 with a 1:1 mixture of (*S*)-**5** and labeled and unlabeled (*R*)-**10^+^·**[B(Ar^F^)_4_]^−^ was done. All intensity ratios are close to 1.0 (Figure 9) so that an isotope effect can be ruled out.

The [I_R_/I_R-_*_d6_*] values for the MS spectra of compounds (*S*)-**5**, (*R*)-**6**, and (*R*)-**7** (Figure 9 and Figures S21–S23) showed a slight isotopic effect (see experimental section for the exact values and the calculation procedures). According to the literature, the directionality of isotope effects (IEs) is difficult to predict from system to system. The literature supports observation of deuterium IEs from both solution and gas phase [44]. Therefore, the observed effects in our systems might be due to decreased van der Waals interactions between the guest’s deuterated moiety and the host; or a preferential ionization of one diastereomeric complex over the other one; or a different gas-phase behavior between the deuterated and non-deuterated guests.

#### 2.4.3. Chiral Discrimination

**Figure 10:**
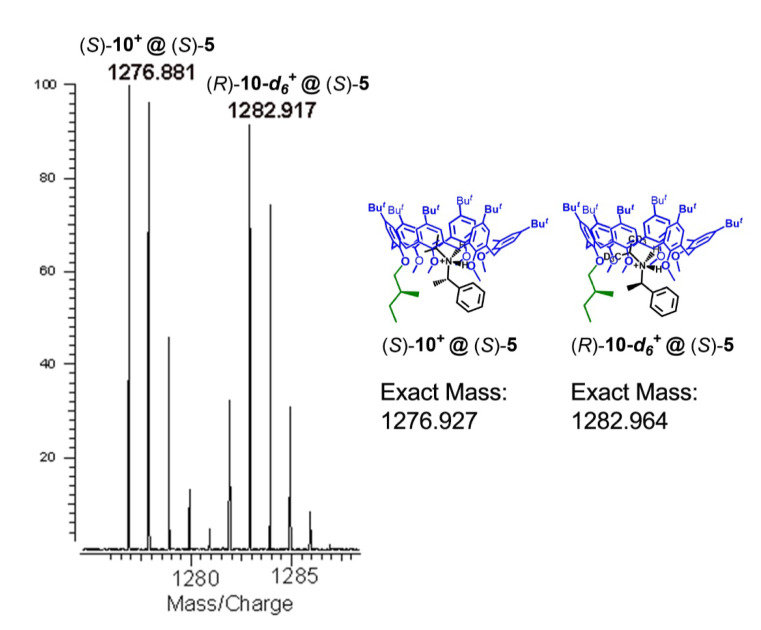
Significant portion of the mass spectrum of a 1:2:2 mixture (CH_2_Cl_2_, 300 μM) of derivatives (*S*)-**5**, (*S*)-**10^+^**·[B(Ar^F^)_4_]^−^, and (*R*)-**10**-*d_6_***^+^**·[B(Ar^F^)_4_]^−^.

In order to determine a chiral discrimination effect of a 1:1 mixture of a pair of labelled (*R*)-enantiomer, (*R*)-**10**-*d*_6_**^+^**·[B(Ar^F^)_4_]^−^, and unlabelled (*S*)-enantiomer guest, (*S*)-**10^+^**·[B(Ar^F^)_4_]^−^, was used (Figure 10) with 0.5 equivalent of the corresponding host and the I_S_/I_R-_*_d6_* ratio value was measured by inspection of the mass spectrum. The resulting mass spectra for the investigation of the isotopic effect are reported below (Figure 10) and in SI (Figures S24–S26).

For the hosts (*S*)-**5**, (*R*)-**6**, and (*R*)-**7** the I_S_/I_R-d6_ were slightly different from the unit. However, their values were almost identical to those regarding the isotopic effect and, therefore, there was not a clear enantiodiscrimination with the hosts studied (see experimental section for the exact values and the calculation procedures). Hence, the mass spectrometric experiments indicate that no significant differentiation of the two diastereomeric complexes occurs.

### 2.5. Possible Rationales

The above results clearly indicated that, independently by the used technique, the enantiodiscrimination ability of the studied hosts are negligible. This unexpected result could be explained by the scarce reciprocal influence between the guest and host chiral centers. In particular, in the case of the “*endo*-chiral” complexes above defined, it is clear that from the point of view of the guest the cavity appears as “too-symmetric” due to the high conformational mobility of the aromatic walls, which do not give any real steric restriction towards the guest geometry. In analogy with chiral crown-ethers [45,46,47], this is a lack of “central-cavity discrimination”.

In the case of the “*exo*-chiral” complexes the two host/guest chiral centers appear to be close enough to affect each other. The observed lack of influence could be then ascribed to their high reciprocal freedom of movements associated to their scarce supramolecular interactions, which is a lack of “lateral discrimination” [45,46,47]. On the basis of these considerations, we can expect that all the factors able to rigidify the cavity in a fixed asymmetric geometry should improve the “central-cavity discrimination”, while the “lateral discrimination” should be improved by the presence of stereoelectronic complementary interacting groups.

## 3. Conclusions

In this work we report an initial study on the influence of chirality in calixarene threading by exploiting the “superweak anion approach” to give chiral pseudo[2]rotaxanes bearing a classical stereogenic element in their axle and/or wheel components. In a first instance, “*endo*-chiral” pseudo[2]rotaxane stereoadducts, having the stereogenic center of a cationic axle inside the calix-cavity of a chiral calixarene, were studied. Successively, the “*exo*-chiral” ones, with the stereogenic center outside the calix-cavity, were also considered. Both “*endo*-chiral” and “*exo*-chiral” pseudo[2]rotaxane stereoadducts were preferentially formed with specifically designed chiral axles by a fine exploitation of the so-called “*endo*-alkyl rule” and a newly defined “*endo*-α-methyl-benzyl rule” [*threading of a hexaalkoxycalix[6]arene with a directional (α-methyl-benzyl)benzylammonium axle occurs with an* endo*-α-methyl-benzyl preference*]. In both instances, the use of 1D and 2D NMR techniques, to establish if any enantiodiscrimination is occurring, led to no conclusive results because of the large number of partially overlapping signals. The pseudorotaxanes were then studied in the gas-phase by means of mass spectrometry, using the enantiomer-labeled guest method. This required the synthesis of enantiopure host/guest couples, including a pair protiated/deuterated chiral axles. Also in this instance, there was not a clear enantiodiscrimination in the threading process with the studied hosts. Possible rationales were given to explain the scarce reciprocal influence between the guest and host chiral centers, which can be useful for future studies in the chiral threading of classical calixarenes and new macrocyclic arenes.

## 4. Experimental Section

ORD spectra were recorded on a JASCO J600 (Tokyo, Japan) spectropolarimeter at room temperature, in acetonitrile as solvent. During the measurement, the instrument was thoroughly purged with nitrogen. Mass spectra were recorded with a Finnigan Mat 711 (Waltham, MA, USA) (EI, 80 eV, 8 kV), an Agilent 6210 ESI-TOF, and an Agilent QFT-7 FTICR (Santa Clara, CA, USA) mass spectrometer with Micromass Z-Spray ESI source, sample cone 25V, HV 2500 V. Flash chromatography was performed on Merck (Darmstadt, Germany) silica gel (60, 40–63 μm). All chemicals were reagent grade and were used without further purification. Anhydrous solvents were purchased from Aldrich (Darmstadt, Germany). When necessary compounds were dried in vacuo over CaCl_2_. Reaction temperatures were measured externally. Reactions were monitored by TLC on Merck silica gel plates (0.25 mm) and visualized by UV light, or by spraying with H_2_SO_4_-Ce(SO_4_)_2_ or phosphomolybdic acid. 1D NMR spectra were recorded on a Bruker (Billerica, MA, USA) Avance-400 spectrometer [400 (1H) and 100 MHz (13C)], Bruker Avance-300 spectrometer [300 (1H) and 75 MHz (13C)] and Bruker Avance-250 spectrometer [250 (1H) and 63 MHz (13C)]; chemical shifts are reported relative to the residual solvent peak (CHCl_3_: δ 7.26, CDCl_3_: δ 77.23; CD_3_OH: δ 3.31, CD_3_OD: δ 49.0). Derivatives **11**, **12**, and **13** [18,19] were synthesized according to literature procedures.

**Synthesis of derivative** (*R/S*)-**4.** NaH (0.072 g, 3.0 mmol) was added to a solution of derivative **14** (0.31 g, 0.30 mmol) in dry DMF (15 mL) and stirred for 1 h at 0 °C. The mixture was allowed to cool at 25 °C, then benzylbromide (0.17 g, 1.00 mmol) was added. The resulting mixture was stirred at 80 °C for 12 h under nitrogen atmosphere, then the solvent was removed under reduced pressure and the mixture was partitioned between CH_2_Cl_2_ and H_2_O. The organic layer was washed with 1N HCl (30 mL), brine (30 mL), and dried over Na_2_SO_4_. The crude product was purified by column chromatography (SiO_2_; CH_2_Cl_2_) to give (0.16 g, 0.14 mmol) of (*R/S*)-**4** as a white solid. Mp: 190–193 °C dec. **ESI(+) MS**: *m*/*z* =1147.81 (MH^+^); **^1^H NMR** (600 MHz, CDCl_3_, 298 K): *δ* 7.47–6.71 (overlapped, 17H, Ar*H_calix_* + ArH*_Bn_*), 5.02 (q, *J* = 6.1, 1H, C*H*), 4.62 and 3.59 (AX, *J* = 13.4 Hz, 2H, ArC*H*_2_Ar), 4.18 and 3.55 (AX, *J* = 14.0 Hz, ArC*H*_2_Ar, 2H), 4.24 and 3.66 (AX, *J* = 14.0 Hz, ArC*H*_2_Ar, 2H), 4.11 and 3.75 (AX, *J* = 13.6 Hz, ArC*H*_2_Ar, 2H), 4.09 and 3.79 (AX, *J* = 13.9 Hz, ArC*H*_2_Ar, 2H), 4.10 and 2.97 (AX, *J* = 13.9 Hz, ArC*H*_2_Ar, 2H), 3.34–3.33 (s, overlapped, OC*H*_3_, 6H), 2.70 (s, OC*H*_3_, 3H), 2.47 (s, OC*H*_3_, 3H), 2.30 (s, OC*H*_3_, 3H), 1.66 (d, *J* = 6.1, 3H, C*H_3Bn_*), 1.32 (s, C(C*H*_3_)_3_, 9H), 1.28 and 1.27 (s, C(C*H*_3_)_3_, 9 H, each one), 0.98, 0.96, 0.89 (s, C(C*H*_3_)_3_, 9H, each one).**^13^C NMR** (100 MHz, CDCl_3_, 298 K): *δ* 154.5, 154.47, 154.43, 153.6, 151.3, 145.8, 145.7, 145.4, 143.3, 134.2, 134.0, 133.8, 133.6, 133.6, 133.5, 133.4, 133.4, 128.4, 127.8, 127.1, 126.7, 124.9, 124.3, 124.1, 81.0, 77.4, 60.3, 60.2, 60.0, 59.9, 34.3, 34.3, 34.2, 34.1, 32.1, 31.7, 31.4, 31.3, 30.8, 30.6, 29.9, 29.5, 22.9.

**Synthesis of derivative** (*S*)-**5.** NaH (0.024 g, 1.0 mmol) was added to a solution of derivative **14** (0.10 g, 0.10 mmol) in dry DMF (15 mL) and stirred for 1 h. The mixture was allowed to cool at room and (*S*)-(+)-1-iodo-2-methylbutane (0.99 g 0.5 mmol) was added. The resulting mixture was kept at 80 °C for 12 h under a nitrogen atmosphere, then the solvent was removed under reduced pressure and the mixture was partitioned between CH_2_Cl_2_ and H_2_O. The organic layer was washed with 1N HCl (30 mL), brine (30 mL), and dried over Na_2_SO_4_. The crude product was purified by column chromatography (SiO_2_; CH_2_Cl_2_) to give derivative (*S*)-**5** as a white solid (0.87g, 0.078 mmol, 78%). Mp: 200–203 °C. [α]25D = +45 (*c* 2.5 CHCl_3_). **ESI(+) MS**: *m*/*z* =1130.773 [M + NH_4_]^+^; **^1^H NMR** (300 MHz, TCDE, 423 K): *δ* 7.31 (br s, ArH, 2H), 7.17 (br s, ArH, 4H), 6.99 (brs, ArH, 2H), 6.95 (brs, ArH, 2H), 6.92 (br s, ArH, 2H), 4.04 (br s, ArCH_2_Ar, 4H), 4.00 (br s, ArCH_2_Ar, 8H), 3.88–3.74 (m, OC*H_2_*CH(CH_3_)CH_2_CH_3_, 2H), 3.37 (s, OCH_3_, 6H), 3.00 (s, OCH_3_, 3H), 2.79 (s, OCH_3_, 6H), 2.07 (m, OCH_2_C*H*(CH_3_)CH_2_CH_3_, 1H), 1.77 (m, OCH_2_CH(CH_3_)C*H_2_*CH_3_, 2H), 1.38 (s, C(C*H*_3_)_3_, 18H), 1.33 (s, C(C*H*_3_)_3_, 9H), 1.24–0.97 (overlapped, OCH_2_CH(C*H*_3_)CH_2_C*H*_3_, 6H), 1.15 (s, C(C*H*_3_)_3_, 18H), 1.05 (s, C(C*H*_3_)_3_, 9H); **^13^C NMR** (75 MHz, CDCl_3_, 298 K): δ 154.4, 153.6, 152.2, 145.8, 145.6, 133.9, 133.7, 133.6 (2), 133.5, 133.3, 127.6, 127.0, 125.0, 124.2, 60.2, 60.1, 60.0, 36.2, 34.3, 34.2, 32.1, 31.7, 31.4, 30.6, 30.3, 29.9, 29.5, 26.4, 22.9, 16.9, 14.3, 11.7.

**Synthesis of derivative***(R,R)-***6.** NaH (0.05 g, 2.0 mmol) was added to a solution of derivative **14** (0.20 g, 0.20 mmol) in dry DMF (10 mL) and stirred for 1 h. The mixture was allowed to cool at room and (*R,R*)-myrtenyl iodide (0.27 g, 1.00 mmol) was added. The resulting mixture was stirred at 80 °C for 12 h under a nitrogen atmosphere, then the solvent was removed under reduced pressure and the mixture was partitioned between CH_2_Cl_2_ and H_2_O. The organic layer was washed with 1N HCl (30 mL), brine (30 mL), and dried over Na_2_SO_4_. The crude product was purified by column chromatography (SiO_2_; CH_2_Cl_2_) to give derivative (*R*)-**6** as a white solid (0.16 g, 0.14 mmol, 75%). Mp: 190–194 °C dec. [α]25D = −15 (*c* 2.5 CHCl_3_). **ESI(+) MS**: *m*/*z* =1194.781 [M + NH_4_]^+^, **^1^H NMR** (300 MHz, TCDE, 353 K): *δ* 7.28 (br s, ArH, 2H), 7.17–7.15 (overlapped, ArH, 4H), 6.90 (br s, ArH, 2H), 6.87 (br s, ArH, 2H), 6.84 (br s, ArH, 2H), 5.79 (br s, C=CH, 1H), 4.84 and 3.51 (AX, *J* = 14.1 Hz, ArC*H*_2_Ar, 4H), 4.30 (br s OC*H*_2_, 2H), 4.27 and 3.66 (AX, *J* = 15.2 Hz, ArC*H*_2_Ar, 4H), 4.16 and 3.78 (AX, *J* = 14.9 Hz, ArC*H*_2_Ar, 2H), 3.38 (s, OCH_3_, 3H), 3.36 (s, OCH_3_, 3H), 2.77 (s, OCH_3_, 3H), 2.63 (s, OCH_3_, 3H), 2.58 (s, OCH_3_, 3H), 2.53–2.20 (4H), 1.40 (s, CH_3_, 3H), 1.38 (s, C(C*H*_3_)_3_, 18H), 1.32 (s, C(C*H*_3_)_3_, 9H), 1.05 (s, C(C*H*_3_)_3_, 18H), 1.00 (s, CH_3_, 3H), 0.97 (s, C(C*H*_3_)_3_, 9H), 0.92 (m, 2H).**^13^C NMR** (75 MHz, CDCl_3_, 298 K) 154.9, 154.2, 152.8, 146.3, 146.2, 145.4, 134.5, 134.4, 134.2, 134.1, 133.8, 127.5, 125.6, 124.8, 120.0, 77.9, 75.9, 60.5, 44.3, 41.6, 38.9, 35.3, 34.9, 34.7, 32.4, 32.2, 31.2, 32.0, 31.9, 31.1, 30.9, 27.0, 25.9, 21.9.

**Synthesis of derivative** (*R*)-**7.** Derivative **14** (0.10 g, 0.10 mmol), DMAP (3.7 mg, 0.030 mmol), and triethylamine (1.0 mL) were mixed with (*S*)-Mosher’s acid chloride (47 mg, 0.20 mmol) in dry DMF (5.0 mL) and the reaction mixture was stirred at 70 °C for 12 h. The reaction mixture was cooled to room temperature and CH_2_Cl_2_ (7 mL) was added. The organic phase was washed with an aqueous solution of HCL (1 N) (3 × 10 mL) and successively with a saturated solution of NaHCO_3_. The organic phase was dried over Na_2_SO_4_, filtered and dried. The crude product was purified by column chromatography (CH_2_Cl_2_: CH_3_OH, 98:2, *v*/*v*) to give derivative (*R*)-**7** as white solid (0.12 g, 0.093 mmol, 93 %). Mp: >210 °C dec. [α]25D = −22 (*c* 2.5 CHCl_3_). **ESI(+) MS**: *m*/*z* =1259.736 [M + NH_4_]^+^; **^1^H NMR** (300 MHz, CDCl_3_, 353 K): δ 7.82 (br m, ArH, 2H), 7.47 (br m, ArH, 3H), 7.24–7.17 (overlapped, ArH, 6H), 6.88–6.83 (overlapped, ArH, 6H), 4.35–4.12 (overlapped, ArCH_2_Ar, 5H), 3.95 (d, ArCH_2_Ar, *J* = 15.4 Hz, 1H), 3.77–3.33 (overlapped, ArCH_2_Ar, 6H), 3.78 (s, OCH_3_, 3H), 3.52 (s, OCH_3_, 3H) 3.40 (s, OCH_3_, 3H), 2.70 (s, OCH_3_, 3H), 2.57 (s, OCH_3_, 3H), 2.47 (s, OCH_3_, 3H), 1.39 (s, C(CH_3_)_3_, 9H), 1.36 (s, C(CH_3_)_3_, 9H), 1.33 (s, C(CH_3_)_3_, 9H), 1.03 (s, C(CH_3_)_3_, 9H), 1.02 (s, C(CH_3_)_3_, 9H), 0.94 (s, C(CH_3_)_3_, 9H); **^13^C NMR** (75 MHz, CDCl_3_, 353 K): δ 165.9, 155.3, 155.1, 154.2, 149.5, 146.6, 146.4, 144.1, 134.2, 134.0, 133.9, 133.6, 133.5, 132.8, 132.3, 130.6, 129.3, 128.7, 128.3, 128.2, 128.0, 127.8, 127.7, 125.6, 125.3, 125.2, 125.1, 60.7, 60.6, 56.5, 34.8, 34.7, 32.2, 32.0, 31.8, 31.3, 31.1.

**Synthesis of derivative** (*S*)-**9^+^**∙[B(Ar^F^)_4_]^−^. (*S*)-α-Methylbenzylamine (0.020 mol) was added to benzaldehyde (0.020 mol) in dry CHCl_3_ (2 mL) and the reaction mixture was stirred at room temperature for 2 h to give the imine intermediate in a quantitative yield.

The resulting imine (0.020 mol) was dissolved in dry MeOH (20 mL) under nitrogen atmosphere and NaBH_4_ (0.20 mol) was added at 0 °C and the mixture was allowed to warm at room temperature and stirred for 3 h. The solvent was removed under reduced pressure and the residue partitioned between AcOEt (30 mL) and an aqueous saturated solution of NaHCO_3_ (30 mL). The organic layer was dried over MgSO_4_ and the solvent was removed under reduced pressure, to give derivative **20** as a yellow viscous liquid. The compound was used for the next step without further purification. The crude product (0.010 mol) was dissolved in Et_2_O (20 mL) at room temperature and an aqueous solution of HCl (37% *w*/*w*, 0.02 mol) was added dropwise. The mixture was stirred for 1 h, until the formation of a white precipitate. The solid was collected by filtration, purified by crystallization with acetonitrile and dried under vacuum, to give derivative **21** as a white solid. Derivative **21** was dissolved in dry MeOH (0.2 M), then NaB(ArF)_4_ (1.1 equiv) was added and the mixture was kept under stirring overnight in the dark. The solvent was removed and deionized water was added, obtaining a brown precipitate that was filtered off and dried under vacuum to give derivatives **9^+^**∙[B(Ar^F^)_4_]^−^. Derivative **9^+^**∙[B(Ar^F^)_4_]^−^: (0.090 g, 0.22 mmol, 95%). [α]25D = −12 (*c* 2.0 MeOH). Mp: >170 °C dec. **ESI(+) MS**: *m*/*z* = 212.15 (M+). **^1^H NMR** (400 MHz, CD_3_OD, 298 K): *δ* 1.65 (d, *J* = 7.1, 3H, CH_3_), 3.85 and 4.05 (AB, *J* = 13.1, 2H), 4.36 (q, *J* = 7.1, 1H), 7.32–7.42 (overlapped, 6H, ArH), 7.56–7.59 (overlapped, 6H, ArH); **^13^C NMR** (100 MHz, CD_3_OD, 298 K) δ 18.3, 49.3, 58.3, 117.1, 120.3, 123.0, 125.7, 127.2, 128.4, 128.6, 128.9, 129.2, 129.3, 129.4, 129.5, 130.9, 134.4, 136.0, 160.8, 161.2, 161.7, 162.2.

**Synthesis of derivative** (*R*)*- or* (*S*)-**10^+^**∙[B(Ar^F^)_4_]^−^. (*R*)- or (S)-α-Methylbenzylamine (0.08 mol) was dissolved in acetone (60 mL) and the mixture was stirred at reflux for 18 h. The reaction mixture was then cooled to room temperature and the excess of ketone was removed under reduced pressure.

The resulting imine (0.080 mol) was dissolved in dry MeOH (20 mL) under nitrogen atmosphere and NaBH_4_ (0.080 mol) was added at 0 °C, then the mixture was allowed to warm at room temperature and stirred for 3 h. The solvent was removed under reduced pressure and the residue partitioned between AcOEt (30 mL) and an aqueous saturated solution of NaHCO_3_ (30 mL). The organic layer was dried over MgSO_4_ and the solvent was removed under reduced pressure, to give derivative (*R*)- *or* (*S*)-**23** as a yellow viscous liquid. The compound was used for the next step without further purification. The crude product (0.010 mol) was dissolved in Et_2_O (20 mL) at room temperature and an aqueous solution of HCl (37% *w*/*w*, 0.08 mol) was added dropwise. The mixture was kept under stirring for 1 h, until the formation of a white precipitate. The solid was collected by filtration, purified by crystallization with *n*-hexane/MeOH and dried under vacuum, to give derivative (*R*)- *or* (*S*)-**24** as a white solid. Derivative (*R*)- *or* (*S*)-**24** was dissolved in dry MeOH, then NaB(ArF)_4_ (1.1 equiv) was added and the mixture was kept under stirring overnight in the dark. The solvent was removed and deionized water was added, obtaining a brown precipitate that was filtered off and dried under vacuum to give derivative (*R*)- *or* (*S*)-**10^+^**∙[B(Ar^F^)_4_]^−^.

Derivative (*S*)-**10^+^**∙[B(Ar^F^)_4_]^−^: (0.087 g, 0.085 mmol, 95%). Mp: >150 °C dec. [α]25D = −22 (*c* 2.0 MeOH). **ESI(+) MS**: *m*/*z* = 164.14 (M+). **^1^H NMR** (400 MHz, CDCl_3_, 298 K): δ 1.24 and 1.29 (d, *J* = 6.1 Hz, CH_3_, 3H each one), 1.66 (d, *J* = 7.1 Hz, CH_3_, 3H), 4.32 (m, C*H*(CH_3_)_2_, 1H), 4.41 (q, *J* = 7.1, 1H, H), 7.22 (br, ArH, 2H), 7.46–7.53 (overlapped, ArH, 7H), 7.69 (br s, ArH, 8H); **^13^C NMR** (100 MHz, CD_3_OD, 298 K) δ 19.1, 19.4, 20.0, 50.4, 57.8, 117.8, 120.7, 123.4, 126.1, 126.5, 128.6, 128.8, 129.0, 129.3, 129.6, 130.6, 131.4, 135.0, 161.1, 161.6,162.1, 162.6.

**Synthesis of derivative** (*R*)-**10**-*d_6_***^+^**∙[B(Ar^F^)_4_]^−^. (*R*)-α-Methylbenzylamine (0.12 g, 0.0010 mol), Ti(*i*-OPr)_4_ (0.28 g, 0.001 mol) and deuterated acetone (0.0030 mol) were mixed together and stirred for 2 h at room temperature.

The resulting mixture was diluted with MeOD (10 mL) under nitrogen atmosphere and NaBH_4_ (0.0020 mol) was added at 0 °C, then the mixture was allowed to warm to room temperature and stirred for 3 h.

The solvent was removed under reduced pressure and the residue partitioned between AcOEt (30 mL) and an aqueous saturated solution of NaHCO_3_ (30 mL). The organic layer was dried over MgSO_4_ and the solvent was removed under reduced pressure, to give derivative (*R*)-**23**-*d_6_* as a yellow viscous liquid. The compound was used for the next step without further purification. The crude product (0.0010 mol) was dissolved in Et_2_O (20 mL) at room temperature and an aqueous solution of HCl (37% w/w, 0.003 mol) was added dropwise. The mixture was kept under stirring for 1 h, until the formation of a white precipitate. The solid was collected by filtration, purified by crystallization with *n*-hexane/MeOH and dried under vacuum, to give derivative (*R*)-**24**-*d_6_* as a white solid. Derivative (*R*)-**24**-*d_6_* was dissolved in dry MeOH (0.2 M), then NaB(ArF)_4_ (1.1 eq) was added and the mixture was kept under stirring overnight in the dark. The solvent was removed and deionized water was added, obtaining a brown precipitate that was filtered off and dried under vacuum to give derivatives (*R*)-**10**-*d_6_*^+^∙[B(Ar^F^)_4_]^−^.

Derivative (*R*)-**10**-*d_6_*^+^∙[B(Ar^F^)_4_]^−^: (0.98 g, 0.00095 mol, 95%). Mp: >160 °C dec**. ESI(+) MS**: *m*/*z* = 170.18 (M+). **^1^H NMR** (400 MHz, CD_3_OD, 298 K): δ 1.64 (d, *J* = 7.1 Hz, 3H), 3.09 (s, 1H), 4.49 (q, *J* = 7.1 Hz, 1H), 7.47–7.59 (overlapped, 17H, Ar*H*). **^13^CNMR** (63 MHz, CDCl_3_, 298 K): δ 149.6, 149.4, 148.4, 148.3, 146.7, 144.6, 143.7, 143.1, 136.6, 132.7, 129.3, 128.6, 127.6, 127.4, 127.2, 127.0, 126.9, 126.4, 126.2, 125.9, 125.6, 78.1, 34.5, 34.2, 34.1, 33.5, 32.9, 31.8.

### 4.1. General Procedure for MS Experiments (Isotopic Effect)

#### 4.1.1. Sample Preparation

Calixarene derivatives (1.9 × 10^−3^ mmol) were dissolved in 0.5 mL of CHCl_3_ (3.8 × 10^−3^ M solution). Then, the appropriate barfate salts, (*R*)-**10^+^**∙[B(Ar^F^)_4_]^−^ (3.8 × 10^−3^ mmol, 7.6 × 10^−3^ M) and (*R*)-**10**-*d_6_***^+^**∙[B(Ar^F^)_4_]^−^ (3.8 × 10^−3^ mmol, 7.6 × 10^−3^ M) were added and the mixture was stirred for 15 min. Then, the solution was diluted to a concentration of 300 μM with CH_2_Cl_2_ before sample injection.

#### 4.1.2. MS Conditions

Sample concentration 300 μM; flow rate 2–4 μL/min; sample cone: 25 V; HV 2500 V; source temperature and temperature of desolvation gas were kept constant at 40 °C, no nebulizer gas was used for the experiments.

#### 4.1.3. I_R_/I_R-dn_ Evaluation

Due to incomplete labeling of the acetone-*d*_6_ used to prepare the axles, a small contribution of the *d*_5_-labeled axle is present. As this can only be the same enantiomer as the corresponding d6-labeled isotopologue, the intensities of both were added.

This operation is valid assuming that no significant differences and isotope effect occur between the partially deuterated compounds (*d*_5_) and the fully deuterated one (*d*_6_).

### 4.2. General Procedure for MS Experiments (Chiral Recognition Effect)

Calixarene derivatives (1.9 × 10^−3^ mmol) were dissolved in 0.5 mL of CHCl_3_ (3.8 × 10^−3^ M solution). Then, the appropriate barfate salts of (*S*)-**10^+^** (3.8 × 10^−3^ mmol, 7.6 × 10^−3^ M) and (*R*)-**10-***d*_6_^+^ (3.8 × 10^−3^ mmol, 7.6 × 10^−3^ M) were added and the mixture was stirred for 15 min. The solution was diluted at the desired concentration with CH_2_Cl_2_ just before the MS analysis.

**MS conditions**: sample concentration 300 μM; flow rate 2–4 μL/min; sample cone: 25 V; HV 2500 V; source temperature and temperature of desolvation gas were kept constant at 40 °C, no nebulizer gas was used for the experiments.

**I_S_/I_R-dn_ evaluation**: The Is/I_R-dn_ ratios were determined as described above.

### 4.3. DFT Calculations

Input structure files for DFT calculations were obtained by molecular modeling with YASARA 20.7.4 program [48]. The force field parameters were generated with the AutoSMILES utility, which employs semiempirical AM1 geometry optimization and assignment of charges, followed by the assignment of the AM1BCC atom and bond types with refinement using the RESP charges, and finally the assignments of general AMBER force field atom types. DFT optimized structures were obtained by calculations at the B97D3/SVP/SVPFIT level of theory implemented in Gaussian 16 suite of programs [49].

## Supplementary Materials

The following are available online. 1D and 2D, ^1^H and ^13^C NMR spectra of compounds **4–7**; ESI-MS spectra of derivatives **5–7**; 1D and 2D NMR spectra of pseudorotaxanes **4@8^+^**, **4@9^+^**, and **4@10^+^**; MS experiments of threading of chiral calixarenes **5–7**; cartesian coordinates of the DFT-optimized structure of pseudorotaxanes.
