# Supplementary material for: Study on the Influence of Chirality in the Threading of Calix[6]arene Hosts with Dialkylammonium Axles
Source: Molecules. 2020 Nov 15;25(22):5323. doi: 10.3390/molecules25225323 (PMC7696445; doi:10.3390/molecules25225323)
Supplement: Supplementary file 1 [file molecules-25-05323-s001.pdf]

Supporting Information

Study on the Influence of Chirality in the Threading of Calix[6]arene Hosts with Dialkylammonium Axles

Carmen Talotta,[1] Gerardo Concilio,[1] Paolo Della Sala,[1] Carmine Gaeta,[1] Christoph A. Schalley,[2,3,*] and Placido Neri[1,*]

Address:

[1] Dipartimento di Chimica e Biologia " A. Zambelli", Università di Salerno, Via Giovanni Paolo II 132, I-84084 Fisciano (Salerno), Italy.

[2] Institut für Chemie und Biochemie, Freie Universität, Arnimallee 20, 14195 Berlin (Germany).

Christoph A. Schalley, mail: c.schalley@fu-berlin.de

Placido Neri, mail: neri@unisa.it

* Corresponding author

Table of contents

Chart S1 3

Synthesis of chiral calixarenes **4-7** 4

1D and 2D NMR spectra of derivative *(S/R)*-**4**. 5-7

^{1}H and ^{13}C NMR spectra of derivative *(S)*-**5.** 8-9

1D and 2D NMR spectra of derivative *(R,R)*-**6** 10-12

1D NMR spectra of derivative *(R)*-**7** 13-16

ESI-MS spectrum of derivatives (*S*)-**5**, (*R,R*)-**6** and (*R*)-**7**. 17

2D COSY spectrum of derivative (*S/R*)-**4** 18

2D HSQC spectrum of derivative (*S/R*)-**4** 19

General procedure for the synthesis of pseudorotaxane. 20

^{1}H NMR spectrum of derivative (*S/R*)-**4@8^{+}** 21

^{1}H NMR spectrum of derivative (*S/R*)-**4@9^{+}** 22

^{1}H NMR spectrum of derivative (*S/R*)-**4@10^{+}** 23

2D COSY spectrum of derivative (*S/R*)-**4@8^{+}** 24

2D HSQC spectrum of derivative *(S/R)*-**4@8^{+}** 25

2D COSY spectrum of derivative (S/R)-**4@9^{+}** 26

MS experiments of Threading of Chiral Calixarenes (*S*)-**5**, (*R,R*)-**6** and (*R*)-**7** 27

General procedure for MS experiments (isotopic effect) 27

General procedure for MS experiments (chiral recognition effect) 29

Cartesian Coordinates of the DFT-optimized structure of pseudorotaxanes 35

Chart S1

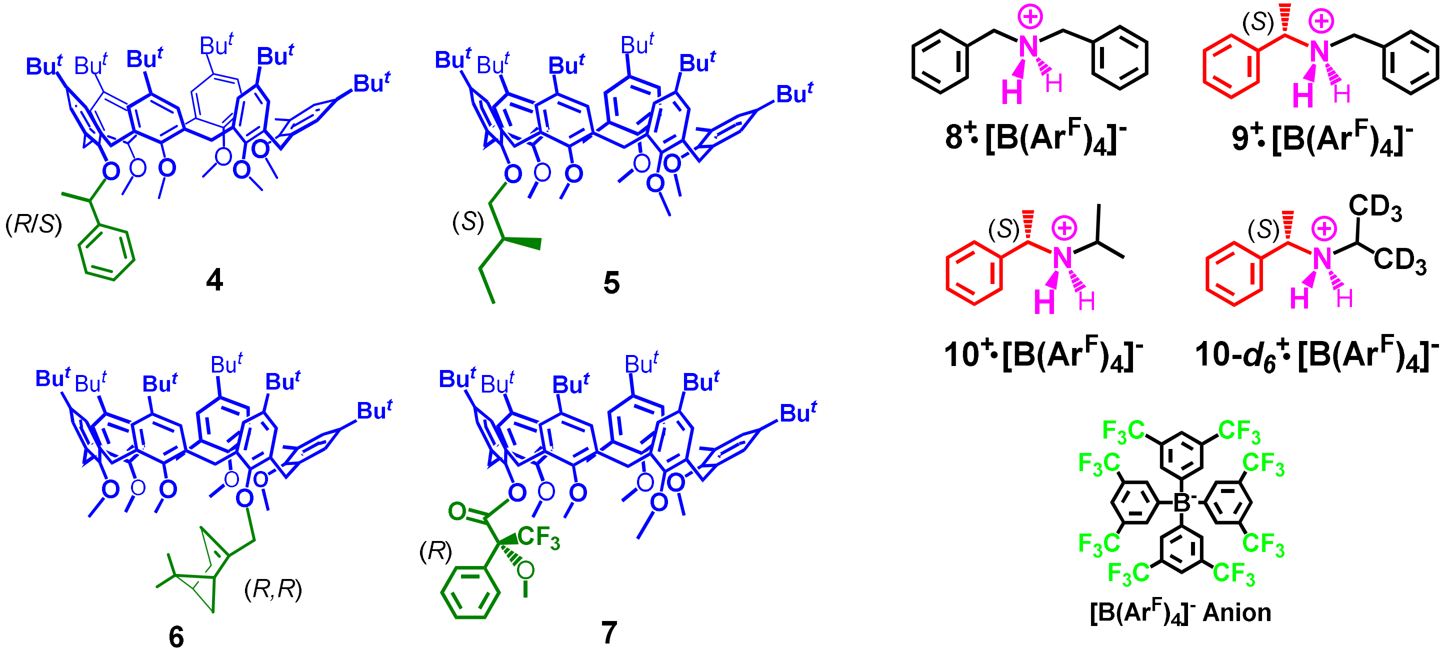

Chart S1. Structures of chiral calix[6]arene hosts **4-7**, alkylammonium axles **8**$^+$**, 9**$^+$**, 10**$^+$**, 10-*d*$_6$**$^+$**,** and $[B(Ar^F)_4]^-$

Synthesis of chiral calixarenes 4-7

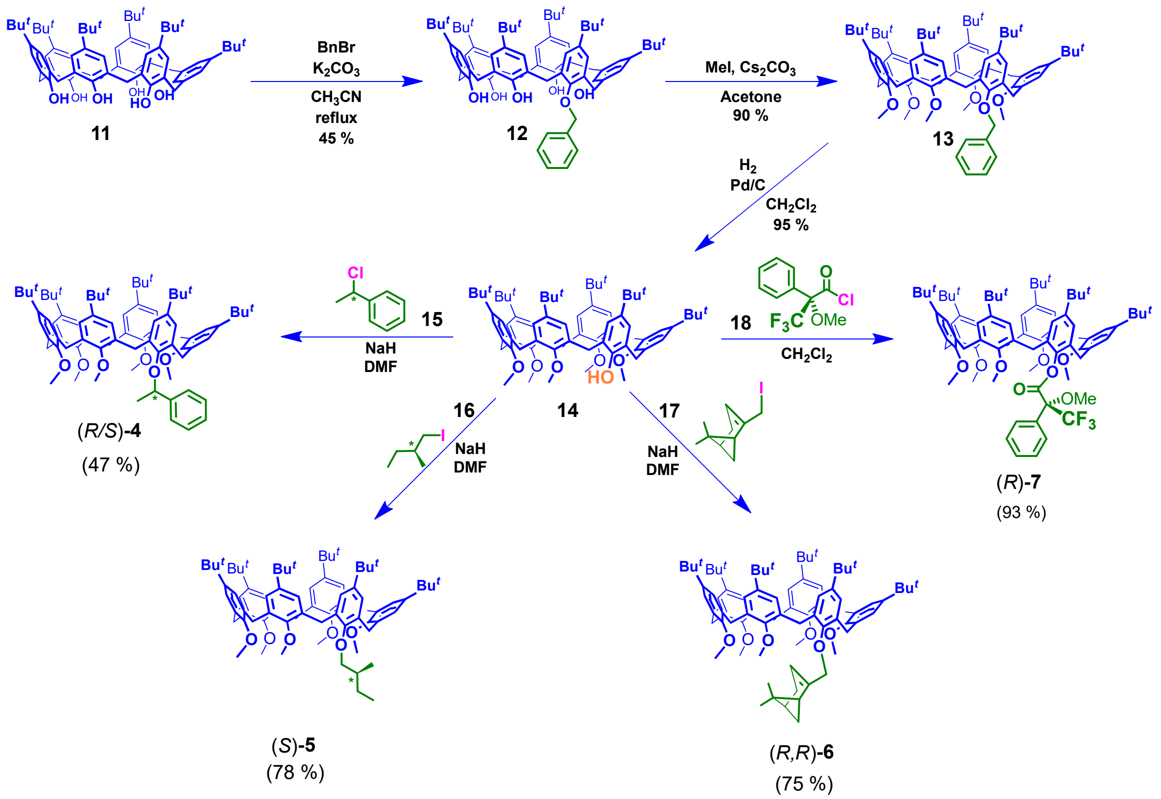

Scheme S1. Synthesis of chiral calixarenes.

1D and 2D NMR spectra of derivative *(S/R)*-4.

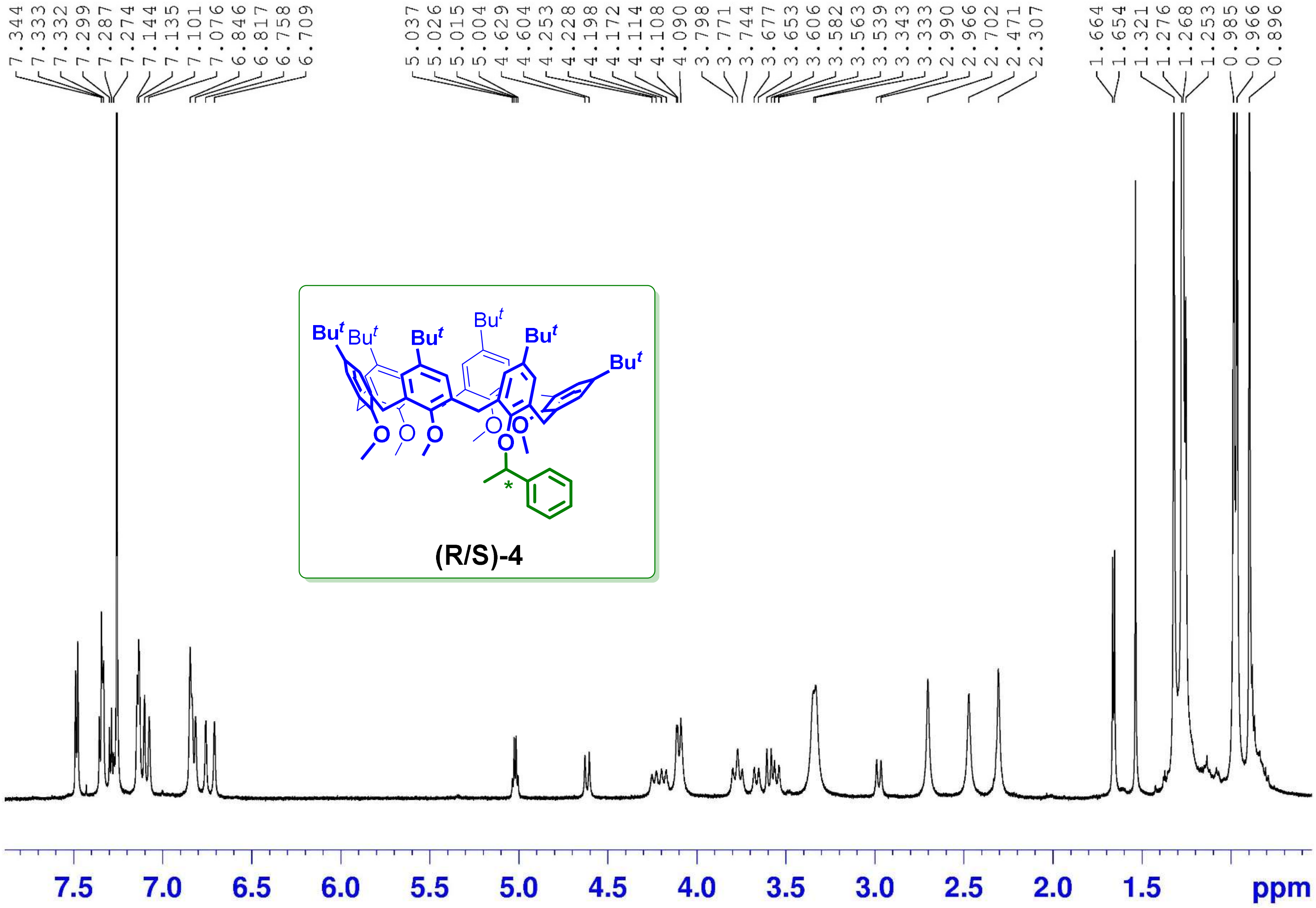

Figure S1. ^{1}H NMR spectrum of derivative (*S*/*R*)-**4** (600 MHz, $CDCl_3$, 298 K).

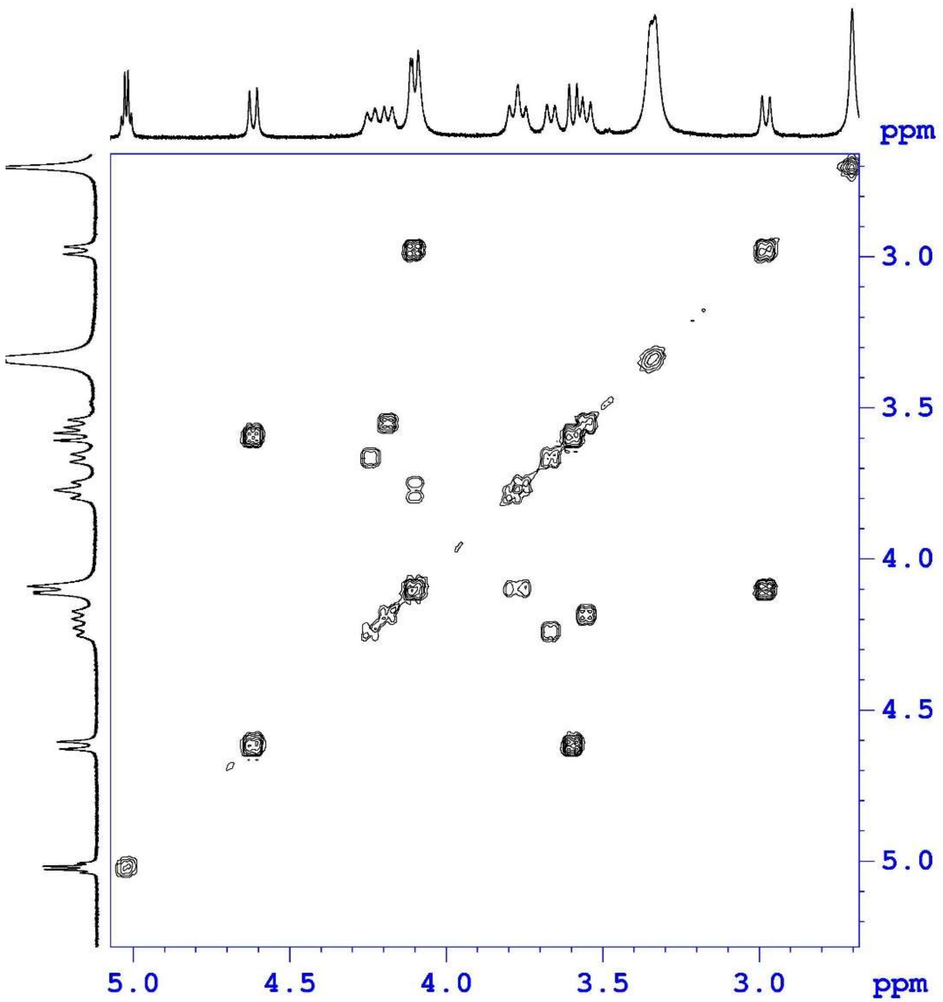

Figure S2. Methylene region of the COSY spectrum of derivative *(S/R)*-**4** (600 MHz, $CDCl_3$, 298 K).

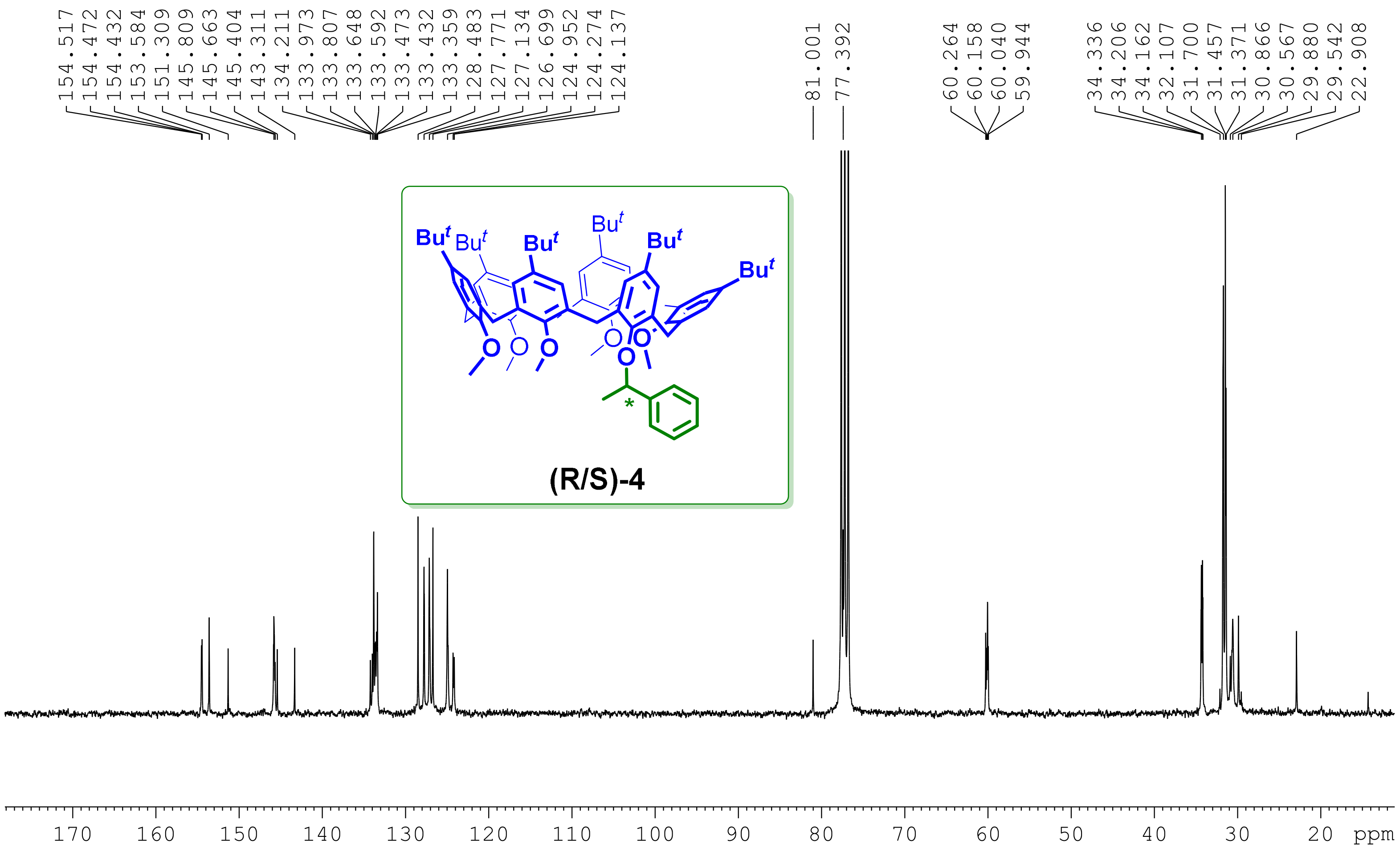

Figure S3. ^{13}C NMR spectrum of derivative (*S*/*R*)-**4** (75 MHz, $CDCl_3$, 298 K).

^{1}H and ^{13}C NMR spectra of derivative *(S)*-5.

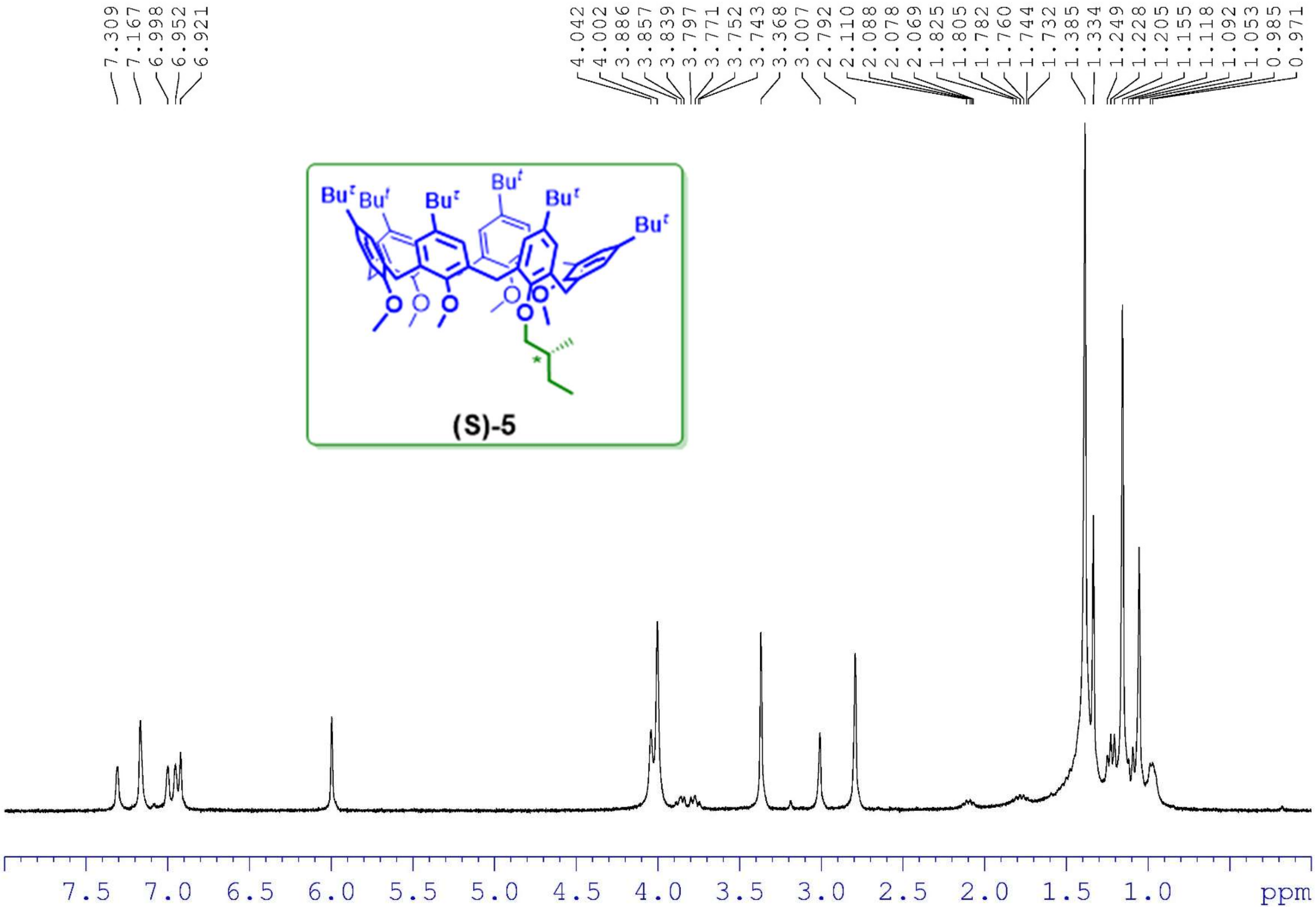

Figure S4. ^{1}H NMR spectrum of derivative *(S)*-**5** (300 MHz, TCDE, 423 K).

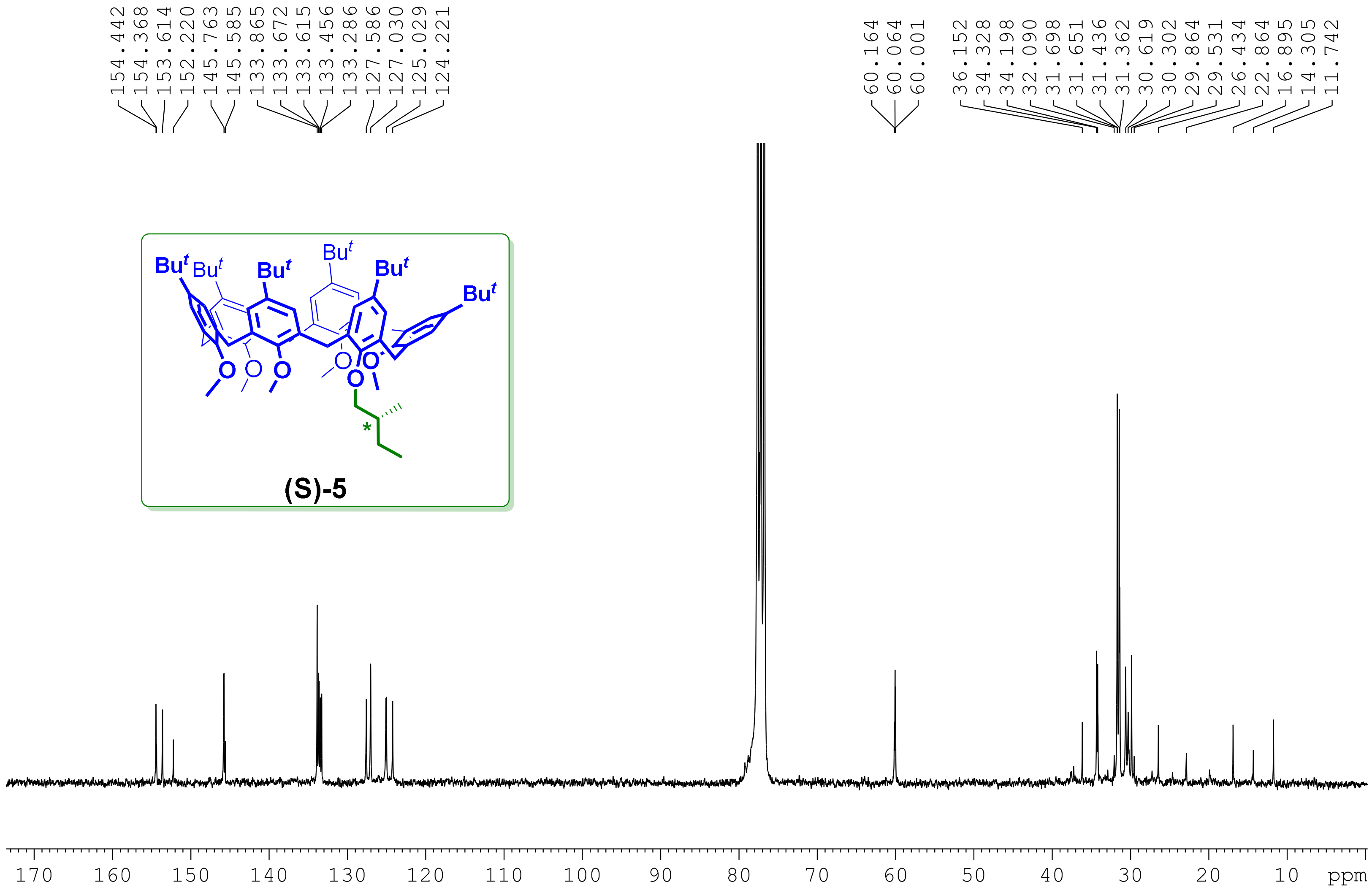

Figure S5. ^{13}C NMR spectrum of derivative *(S)*-**5** (75 MHz, $CDCl_3$, 298 K).

1D and 2D NMR spectra of derivative *(R,R)*-6

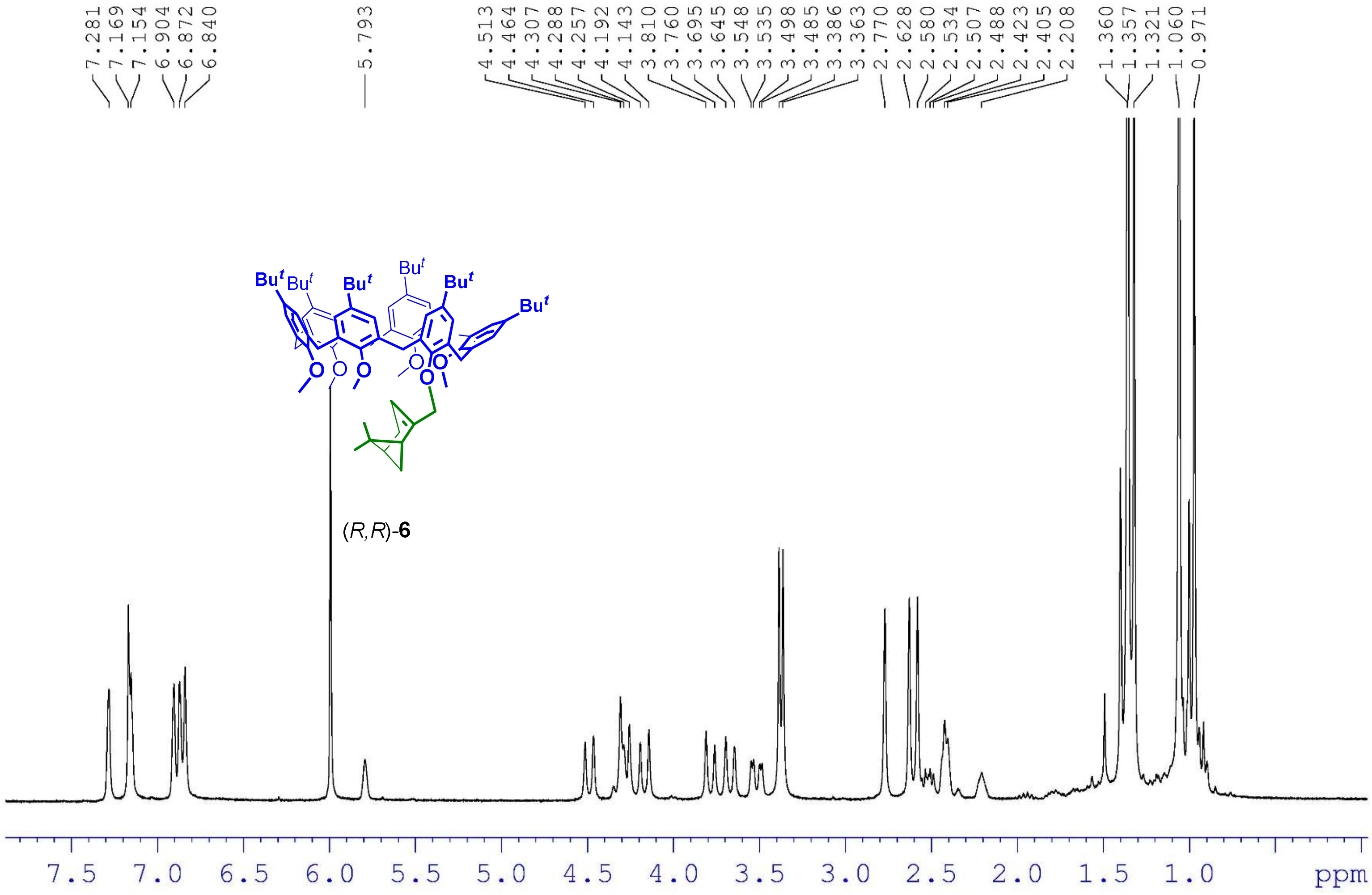

Figure S6. ^{1}H NMR spectrum of derivative *(R,R)*-**6** (600 MHz, TCDE, 353 K).

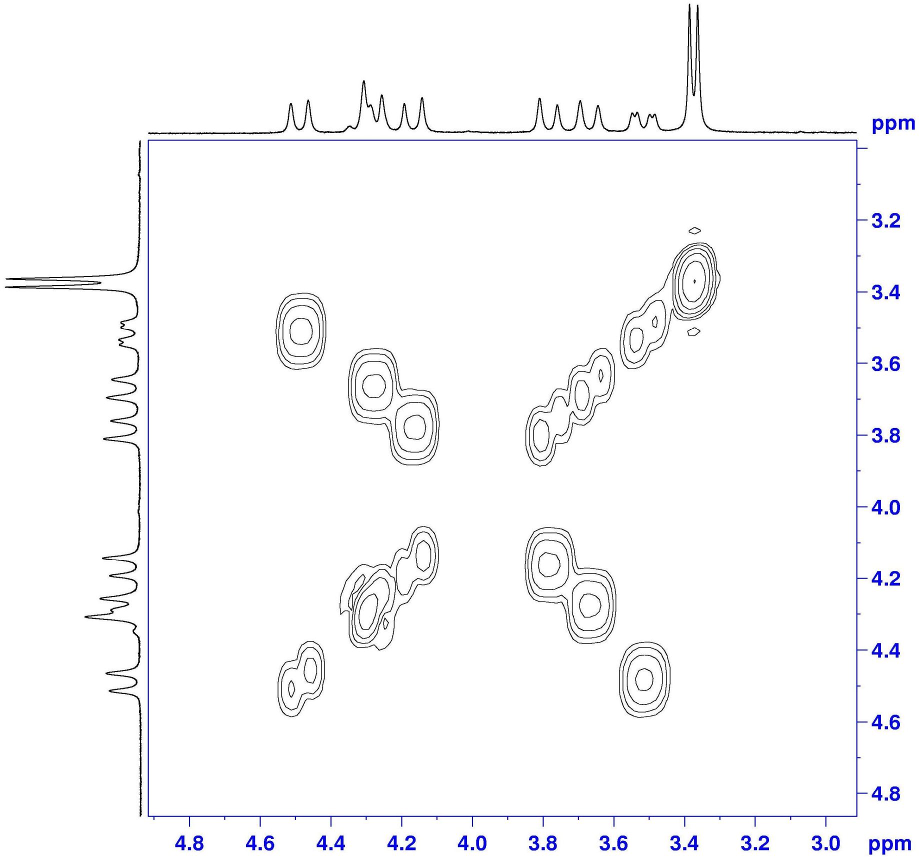

Figure S7. ^{1}H NMR spectrum of derivative *(R,R)*-**6** (600 MHz, TCDE, 353 K).

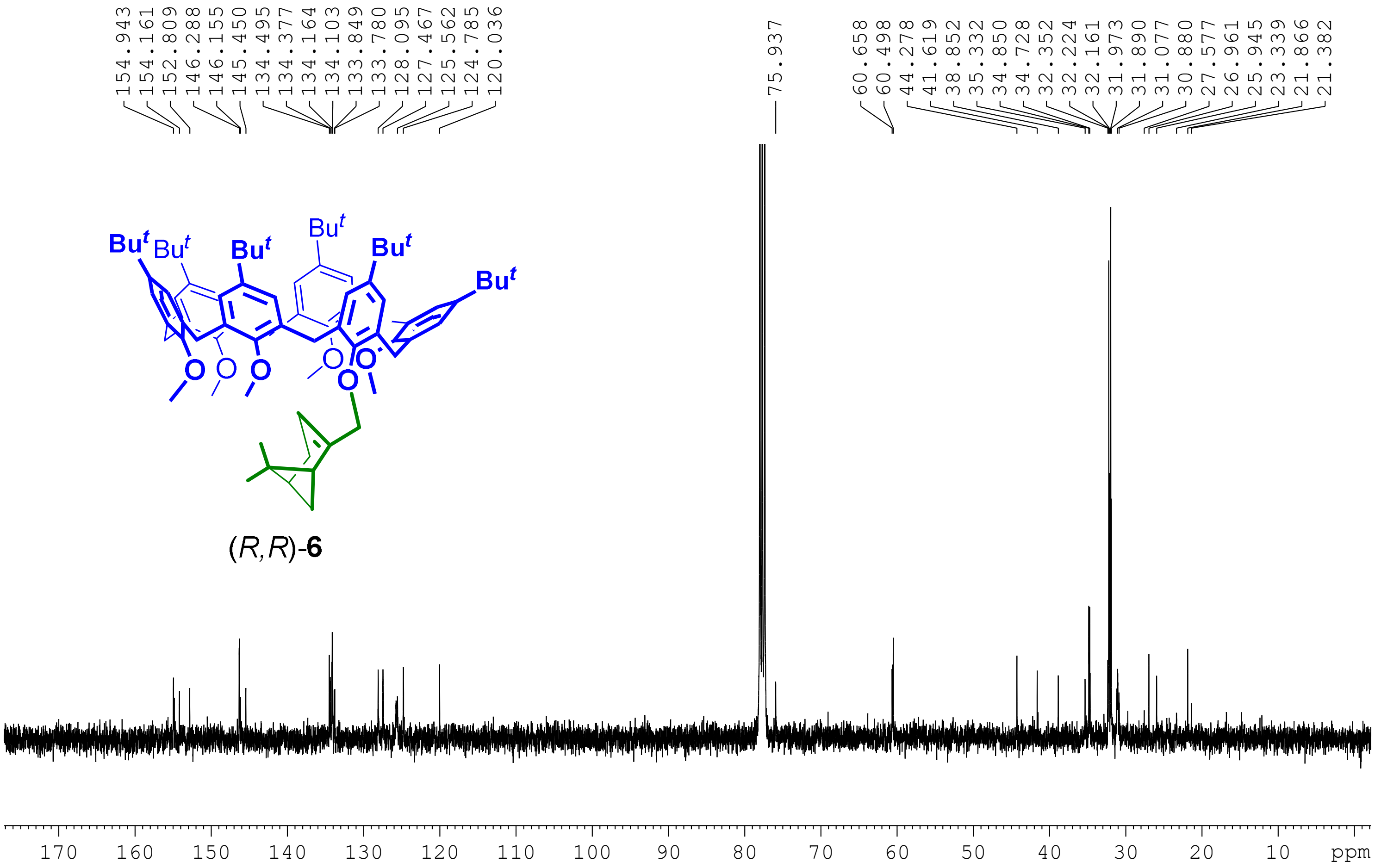

Figure S8. ^{13}C NMR spectrum of derivative (*R*,*R*)-**6** (75 MHz, $CDCl_3$, 298 K).

1D NMR spectra of derivative (*R*)-7

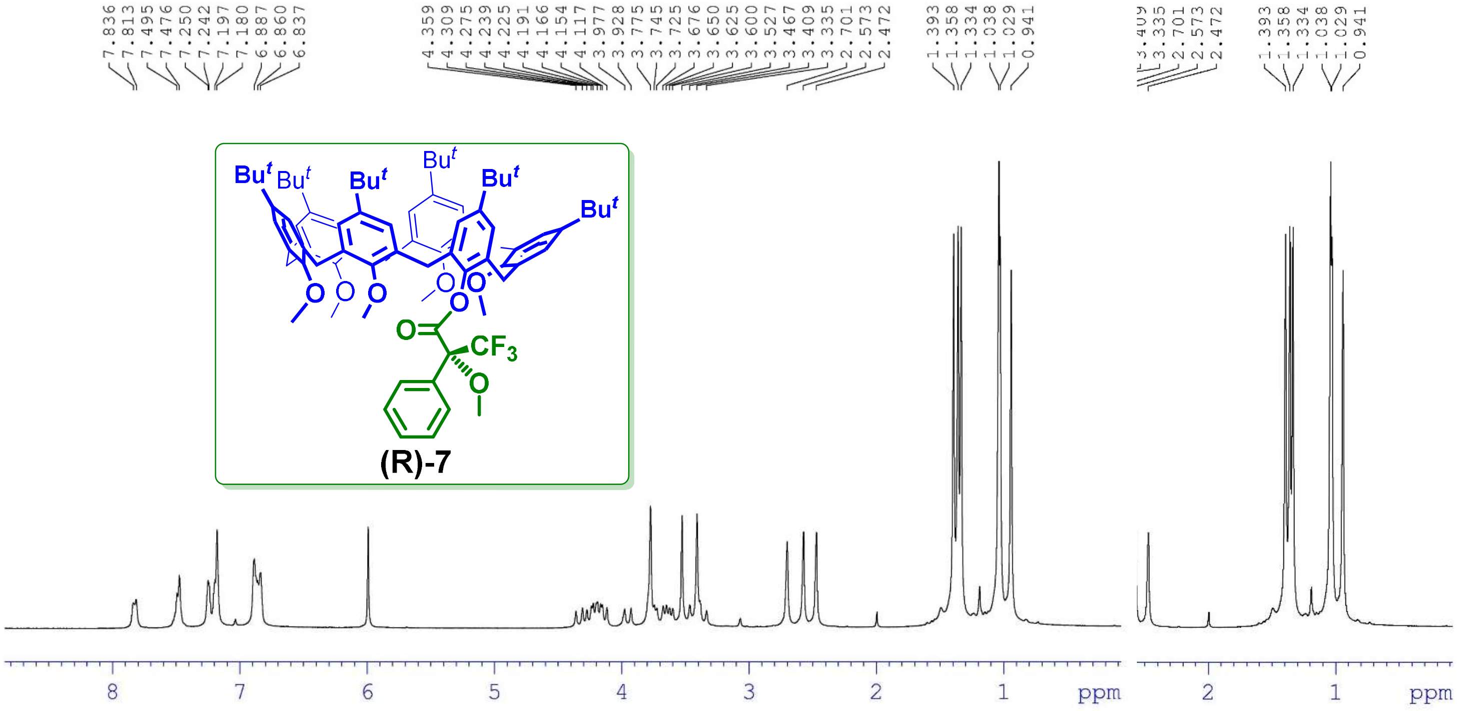

Figure S9. ^{1}H NMR spectrum of derivative *(R)*-**7** (300 MHz, TCDE, 353 K).

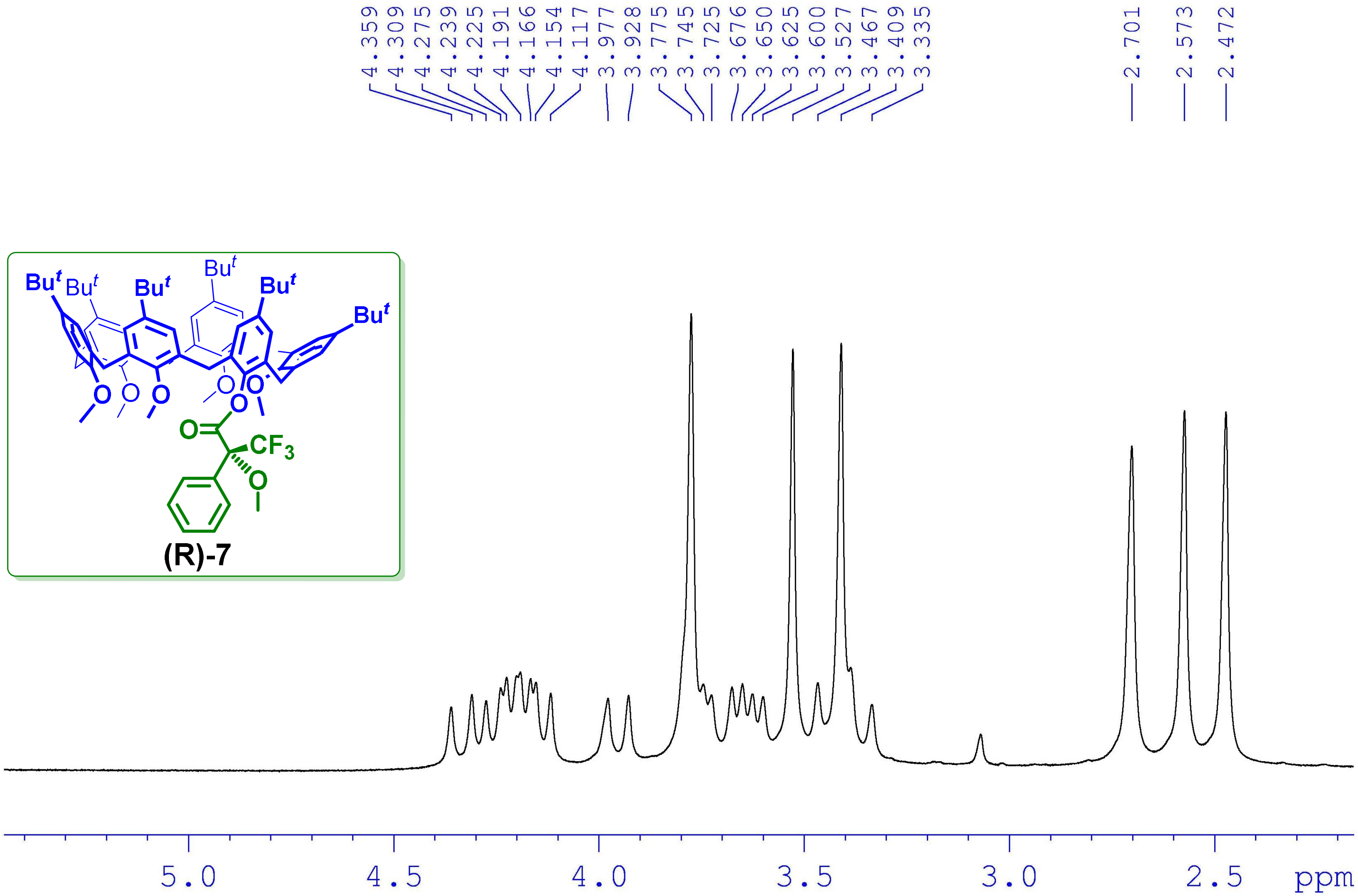

Figure S10. Expansion of the ^{1}H NMR spectrum of derivative *(R)*-**7** (300 MHz, TCDE, 353 K).

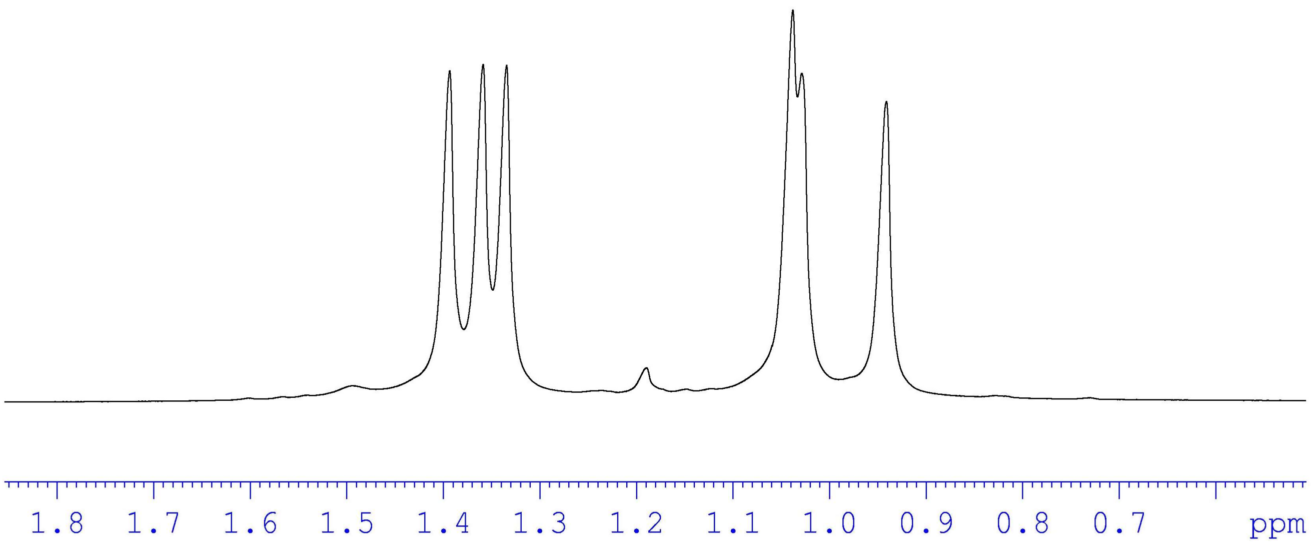

Figure S11. Expansion of the ^{1}H NMR spectrum of derivative *(R)*-**7** (300 MHz, TCDE, 353 K).

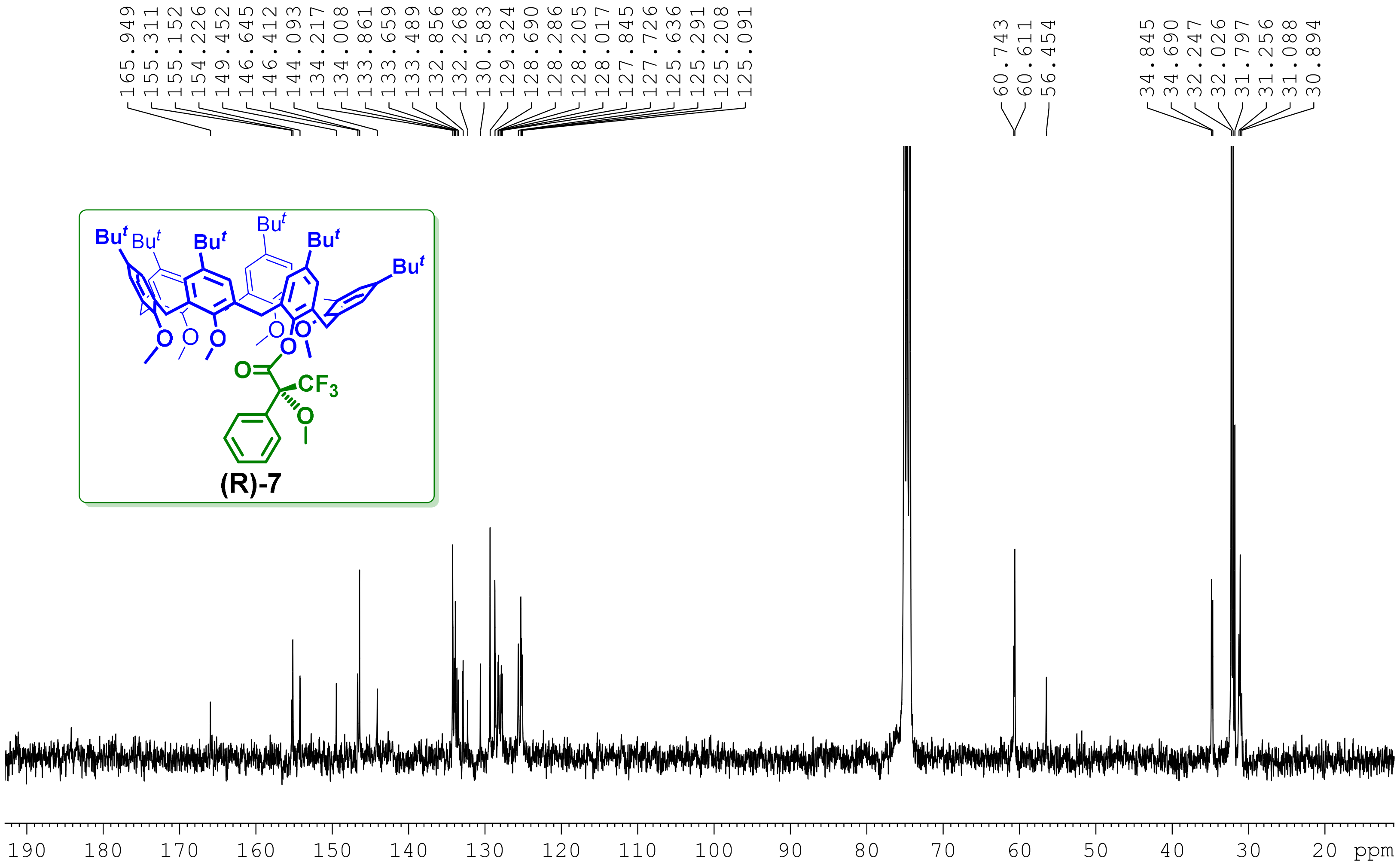

Figure S12. ^{13}C NMR spectrum of derivative (*R*)-**7** (75 MHz, $CDCl_3$, 298 K).

ESI-MS spectra of derivatives (*S*)-5, (*R,R*)-6 and (*R*)-7.

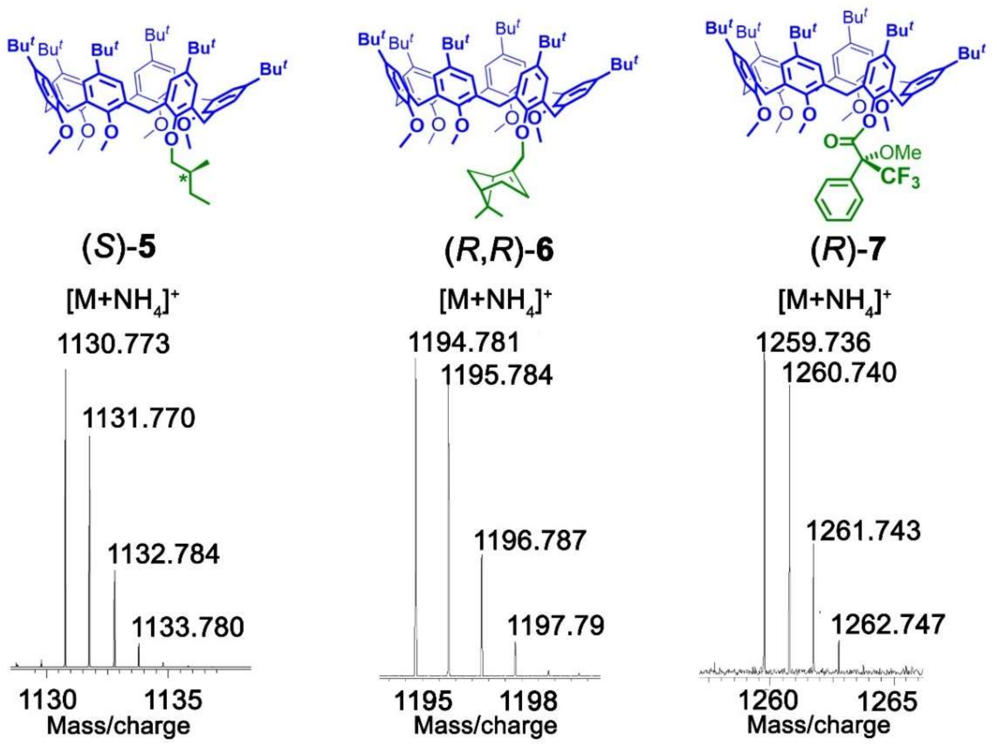

Figure S13. Mass spectra of compounds (*S*)-**5**, (*R*)-**6**, and (*R*)-**7**.

2D COSY spectrum of derivative (*S*/*R*)-4

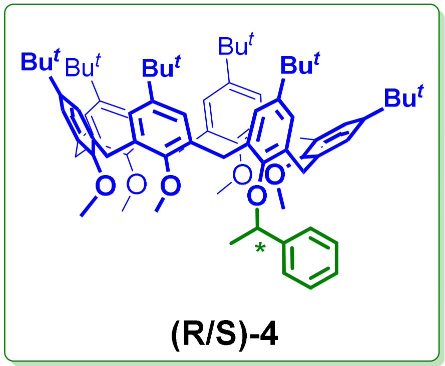

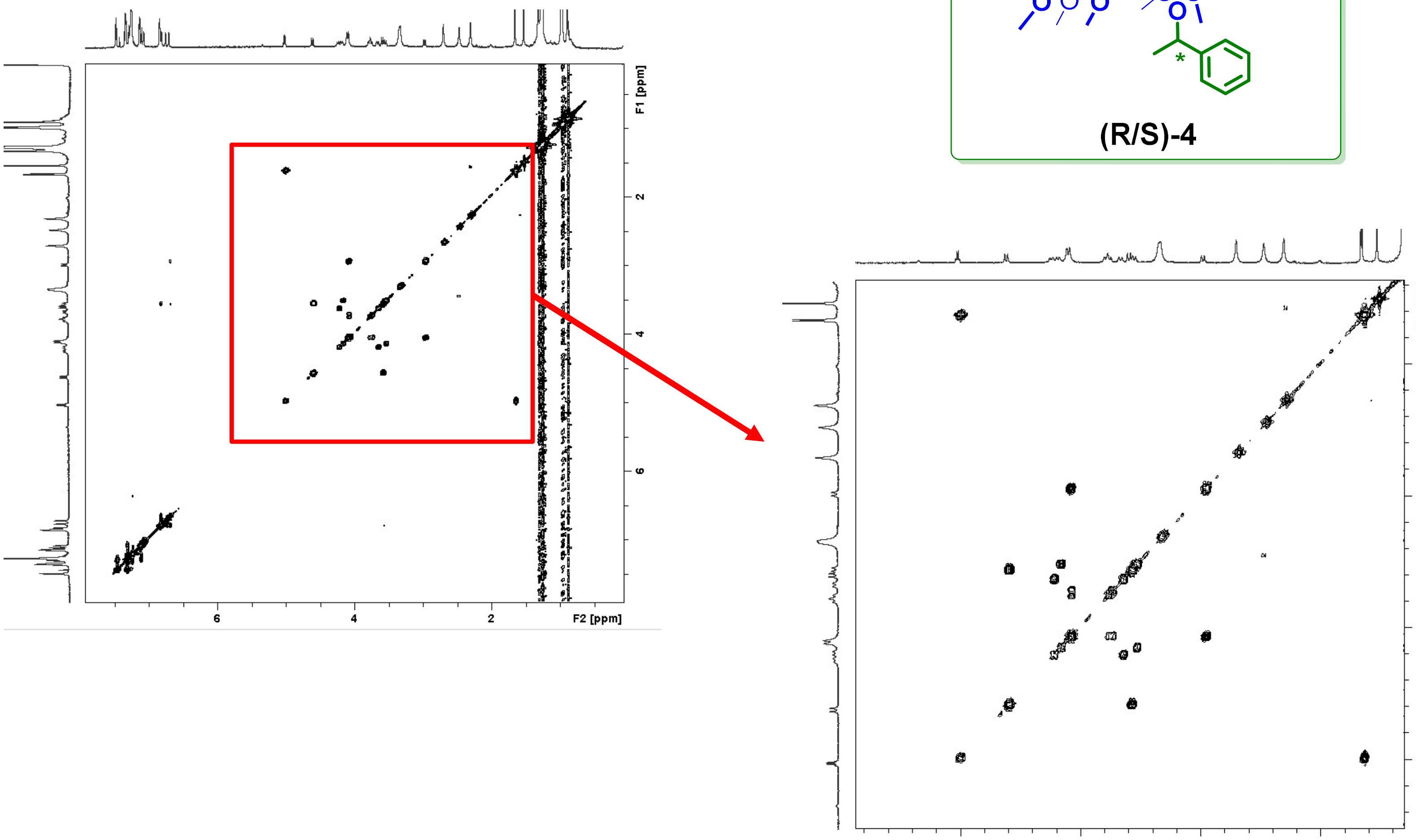

Figure S14. 2D COSY spectrum of derivative (*S*/*R*)-**4** (600 MHz, $CDCl_3$, 298 K).

2D HSQC spectrum of derivative (*S/R*)-4

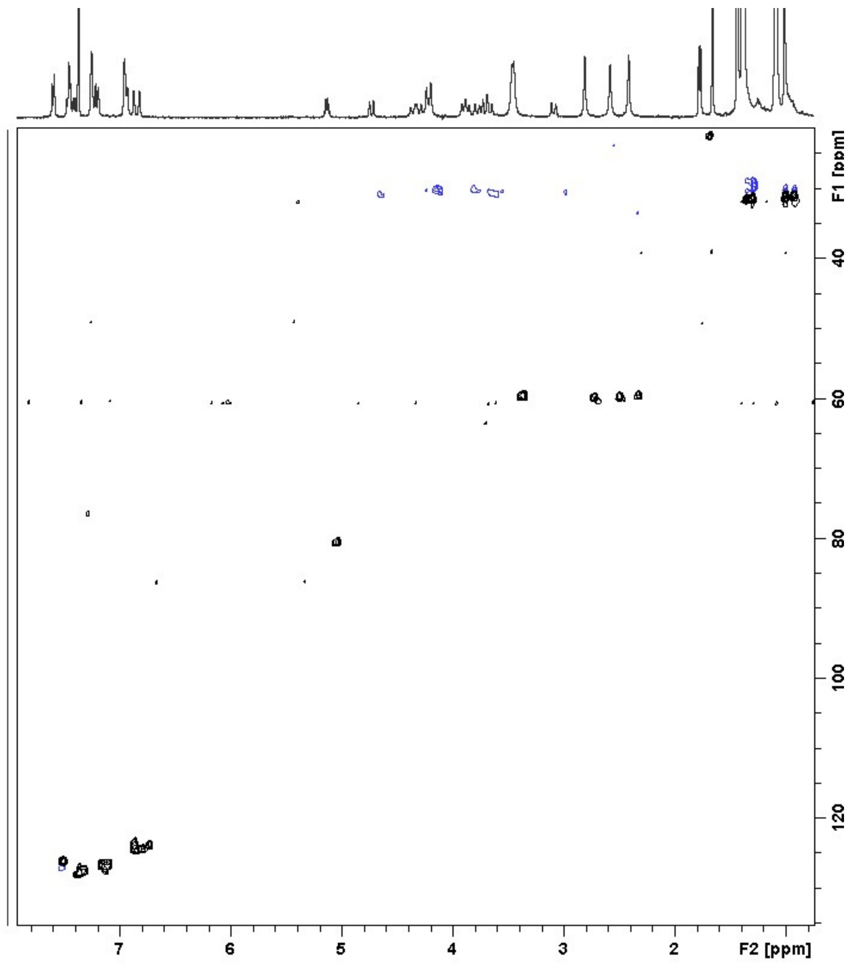

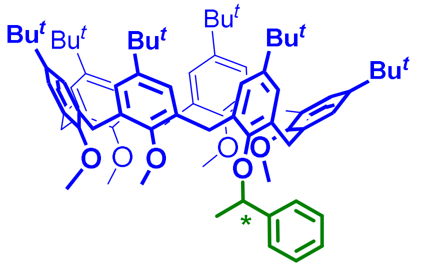

(R/S)-4

Figure S15. 2D HSQC spectrum of derivative (*S/R*)-**4** (600 MHz, $CDCl_3$, 298 K).

General procedure for the synthesis of pseudorotaxanes.

Chiral calixarene derivatives (1.9·10^{-3} mmol) was dissolved in 0.5 mL of $CDCl_3$ (3.8·10^{-3} M solution). Then, the barfate salt was added (1.9·10^{-3} mmol, 3.8·10^{-3} M) and the mixture was stirred for 15 min. Then, the solution was transferred in a NMR tube for 1D and 2D NMR spectra acquisition.

^{1}H NMR spectrum of derivative (*S/R*)-4@8^{+}·[B(Ar^{F})$_4$]$^{-}$

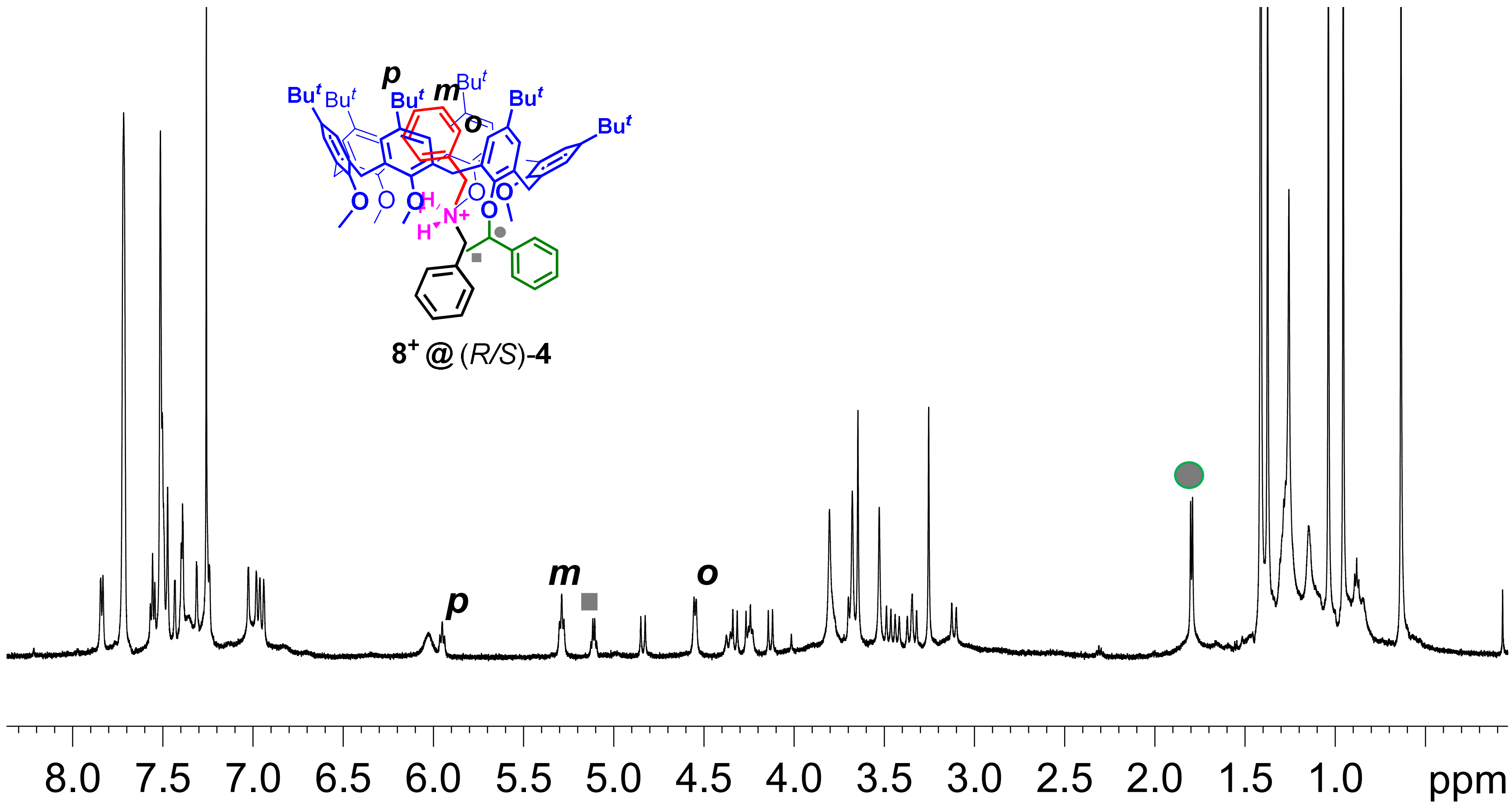

Figure S16. ^{1}H NMR spectrum (600 MHz, $CDCl_3$, 298 K) of 1:1 solution of (*S/R*)-**4** (3.0 mM) and **8**$^{+}$·[B(Ar^{F})$_4$]$^{-}$ (3.0 mM).

^{1}H NMR spectrum of derivative (*S/R*)-4@9$^+$·[B(Ar^F)$_4$]$^-$

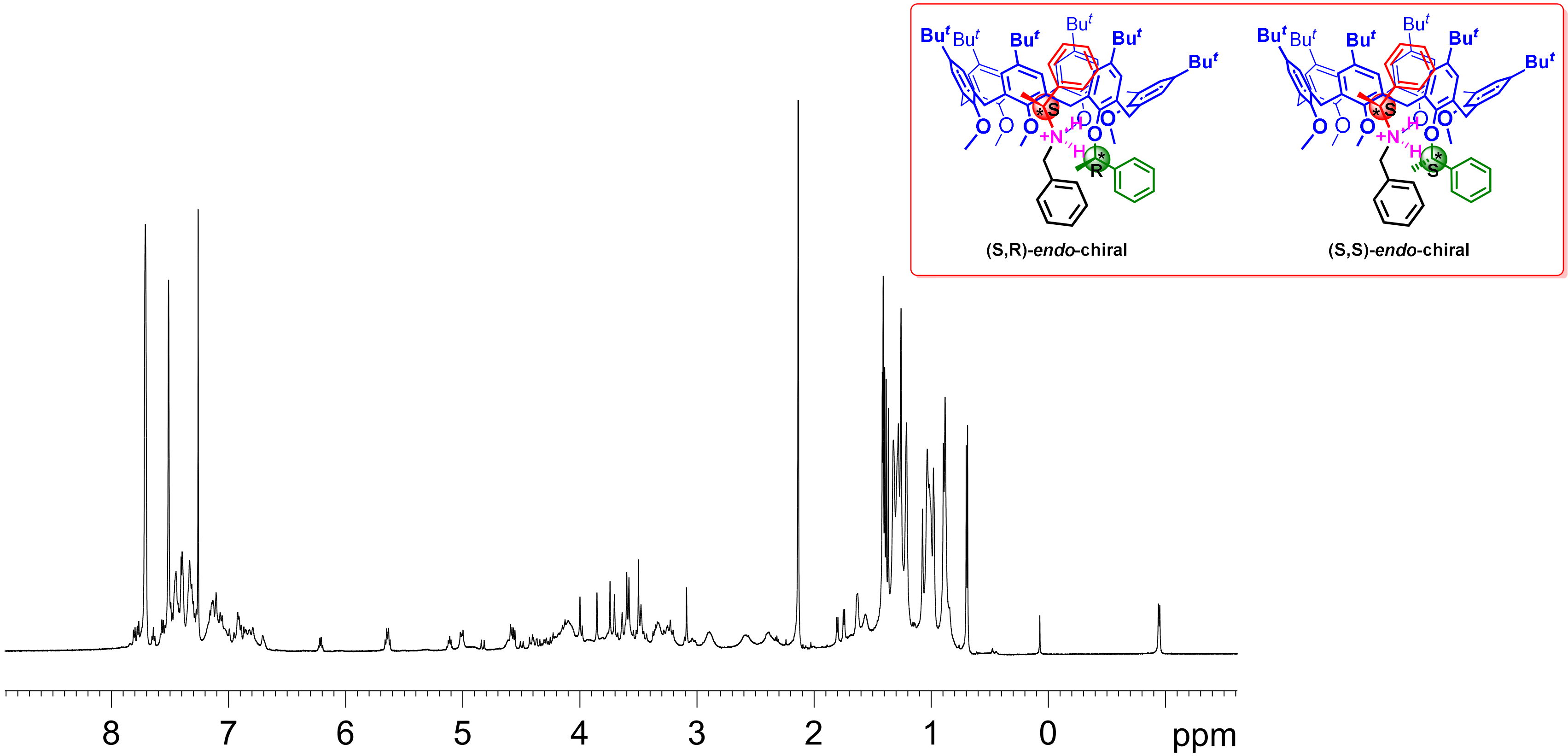

Figure S16. ^{1}H NMR spectrum (600 MHz, $CDCl_3$, 298 K) of 1:1 solution of (*S/R*)-**4** (3.0 mM) and **9**$^+$·[B(Ar^F)$_4$]$^-$ (3.0mM).

^{1}H NMR spectrum of derivative (*S/R*)-4@10$^+$·[B(Ar^F)$_4$]$^-$

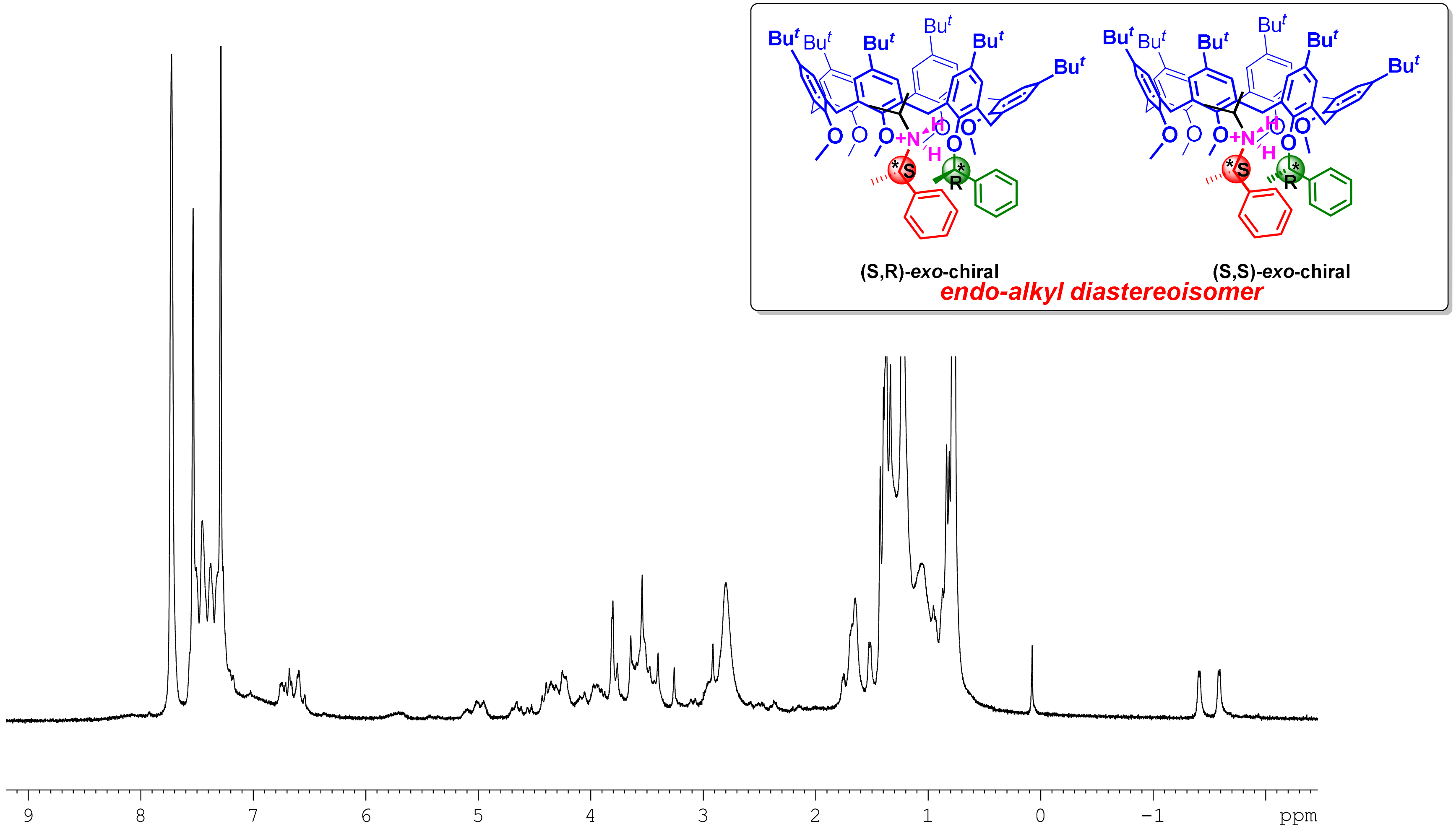

Figure S17. ^{1}H NMR spectrum (600 MHz, $CDCl_3$, 243 K) of 1:1 solution of (*S/R*)-**4** (3.0 mM) and **10**$^+$·[B(Ar^F)$_4$]$^-$ (3.0 mM).

2D COSY spectrum of derivative (*S*/*R*)-4@8^{+}·[B(Ar^{F})$_4$]$^{-}$

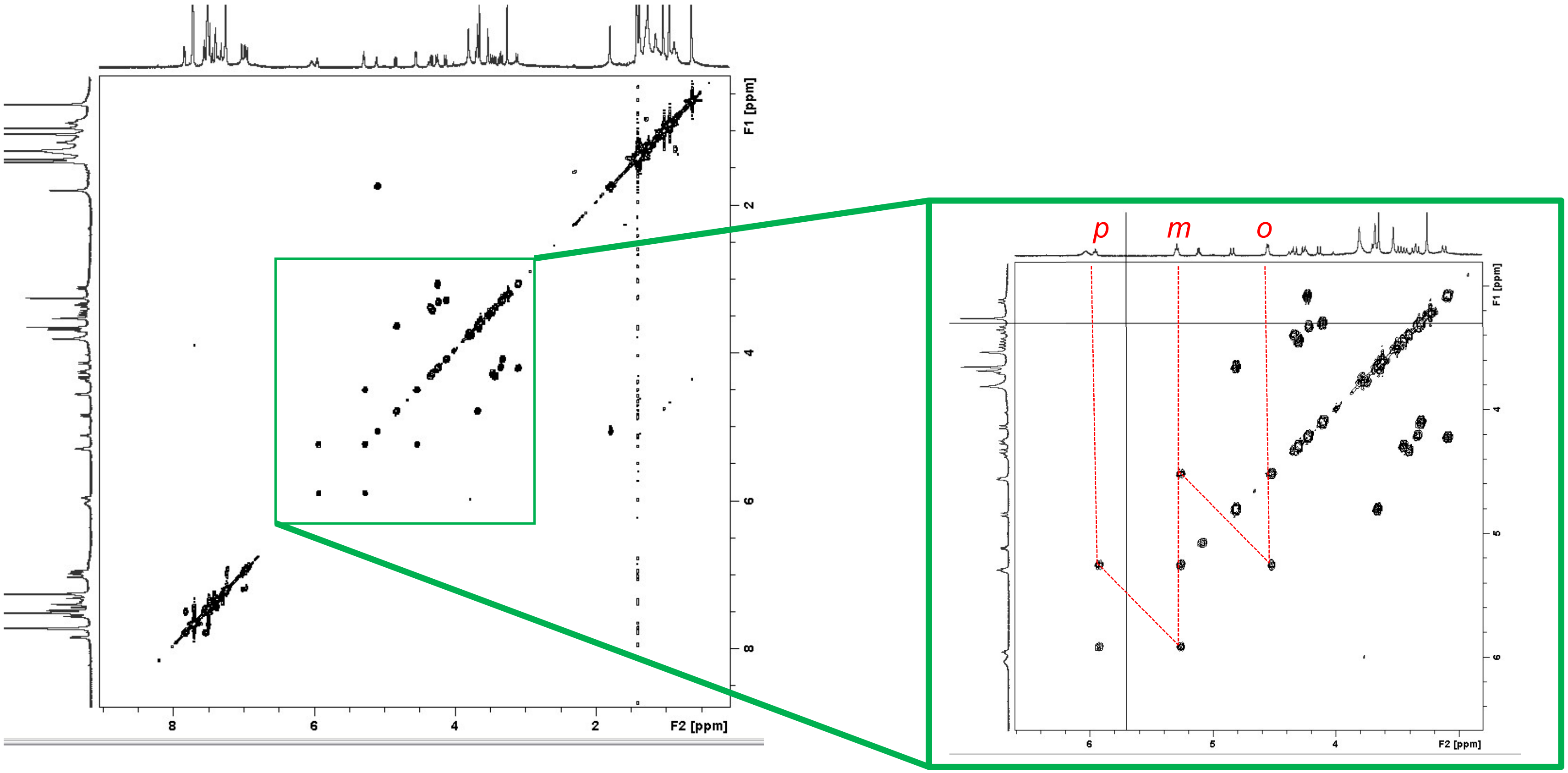

Figure S18. 2D COSY spectrum of derivative (*S*/*R*)-**4@8^{+}**·[B(Ar^{F})$_4$]$^{-}$ ($CDCl_3$, 600 MHz, 298 K).

2D HSQC spectrum of derivative (*S*/*R*)-4@8$^+$·[B(Ar^F)$_4$]$^-$

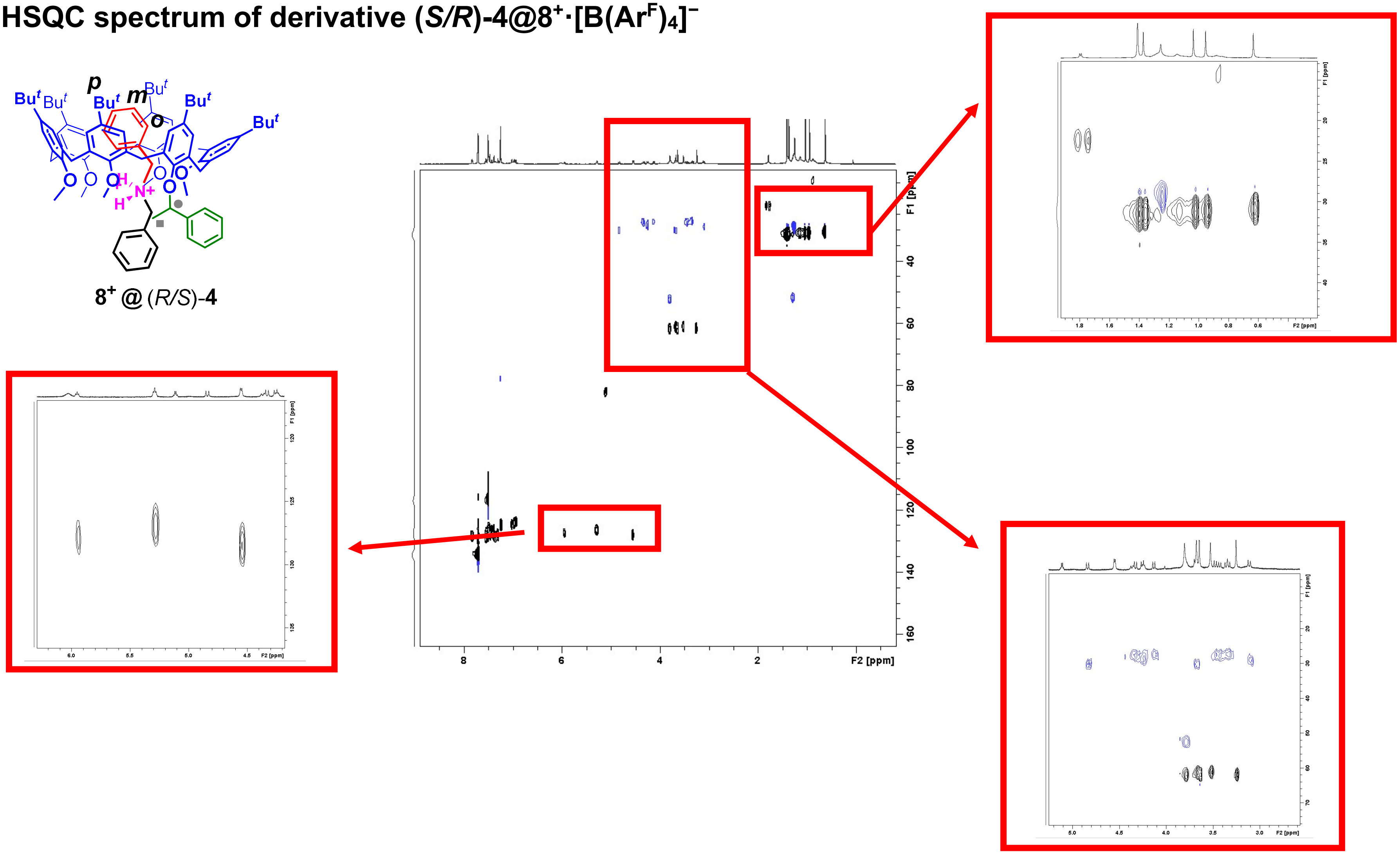

Figure S19. 2D HSQC spectrum and its significant portions, of derivative (*S*/*R*)-**4@8**$^+$·[B(Ar^F)$_4$]$^-$ (600 MHz, $CDCl_3$, 298 K).

2D COSY spectrum of derivative (*S*/*R*)-4@9^{+}·[B(Ar^{F})$_4$]$^{-}$

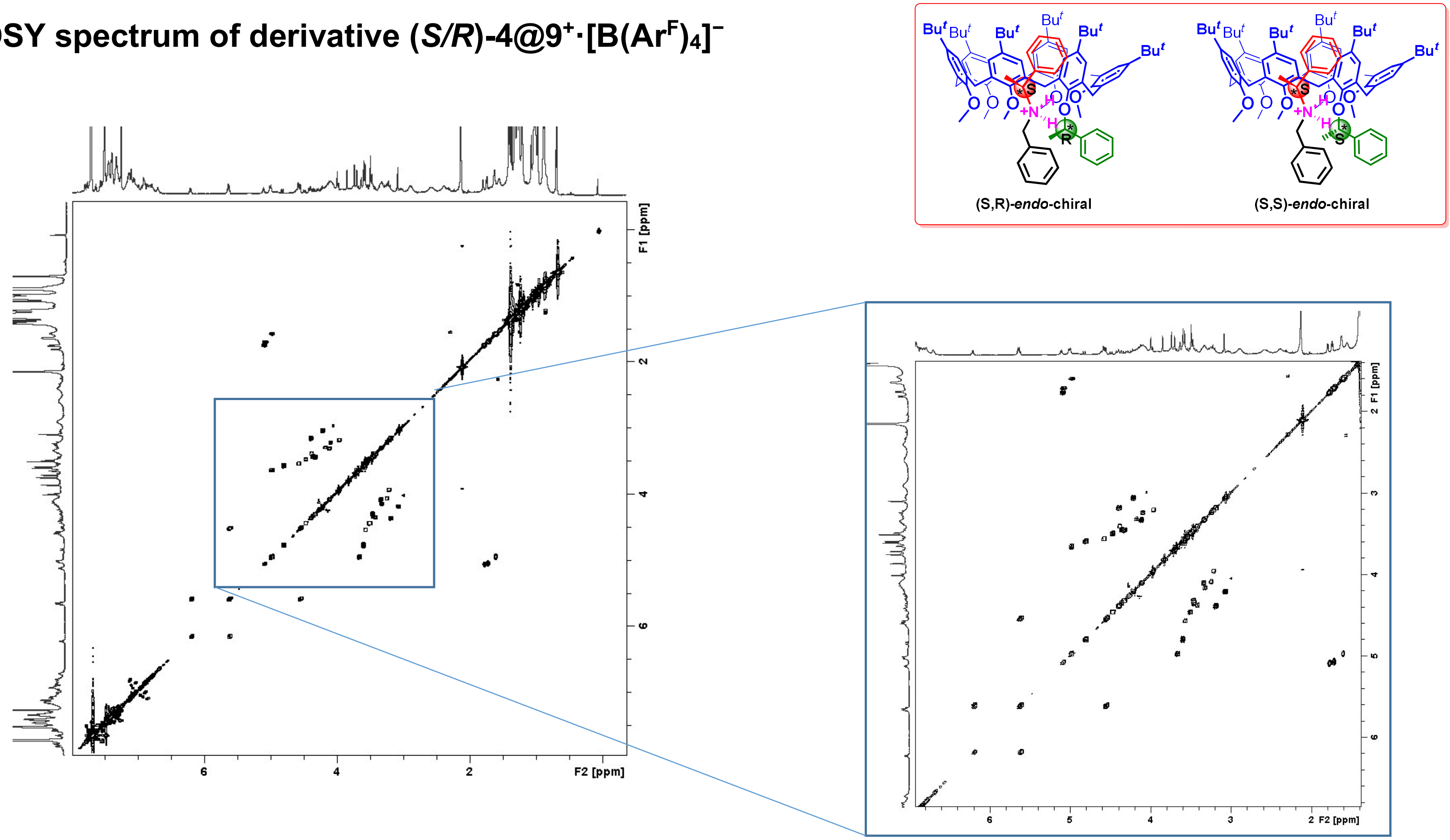

Figure S20. 2D COSY spectrum of derivative (*S*/*R*)-**4@9^{+}**·[B(Ar^{F})$_4$]$^{-}$ (CDCl$_3$, 600 MHz, 298 K).

MS experiments on the Threading of Chiral Calixarenes (*S*)-**5**, (*R*,*R*)-**6**, and (*R*)-**7**

General procedure for MS experiments (isotopic effect)

Sample preparation

The chiral (enantiopure) calixarene derivative ($1.9\cdot10^{-3}$ mmol) was dissolved in 0.5 mL of $CHCl_3$ ($3.8\cdot10^{-3}$ M solution). Then, the barfate salt (*R*)-$\mathbf{10^{+}}\cdot[B(Ar^F)_4]^-$ ($3.8\cdot10^{-3}$ mmol, $7.6\cdot10^{-3}$ M) and (*R*)-**10**-$\boldsymbol{d_6}^{\mathbf{+}}\cdot[B(Ar^F)_4]^-$ ($3.8\cdot10^{-3}$ mmol, $7.6\cdot10^{-3}$ M) were added and the mixture was stirred for 15 min. Then, the solution was diluted at a concentration of 300 µM with CH_2Cl_2 before the MS injection.

MS conditions

Sample concentration 300 µM; flow rate 2-4 µL/min; sample cone: 25 V; HV 2500 V; source temperature and temperature of desolvation gas were kept constant at 40 °C, no nebulizer gas was used for the experiments.

$I_R/I_{R\text{-}d_n}$ evaluation

The d_6-labeled axle is accompanied by a fraction of d_5- and (very minor) d_4-labeled analogues as the deuterated reagent used in the synthesis was not 100% d_6-labeled. As these isotopologues have the same stereochemistry as the d_6-labeled axle, the signal intensities arising from them have been added to those of the d_6-labeled complex.

This operation is valid assuming that no significant differences and isotopic effect occur between the partially deuterated compounds (d_4 and d_5) and the fully deuterated one (d_6). The intensity of the non-deuterated compounds, is than evaluated as the sum of each peak related to its distribution.

The corresponding peak intensities are pasted from the Omega software controlling the mass spectrometer to the spreadsheet, which calculates the intensity of each peak.

Selected examples

1:2:2 mixture of (*S*)-**5**, (*R*)-$\mathbf{10^+}$ ·$[B(Ar^F)_4]^-$ and (*R*)-**10**-d_6^{+}·$[B(Ar^F)_4]^-$

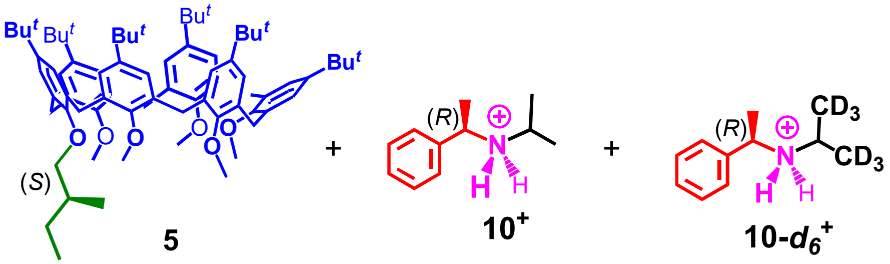

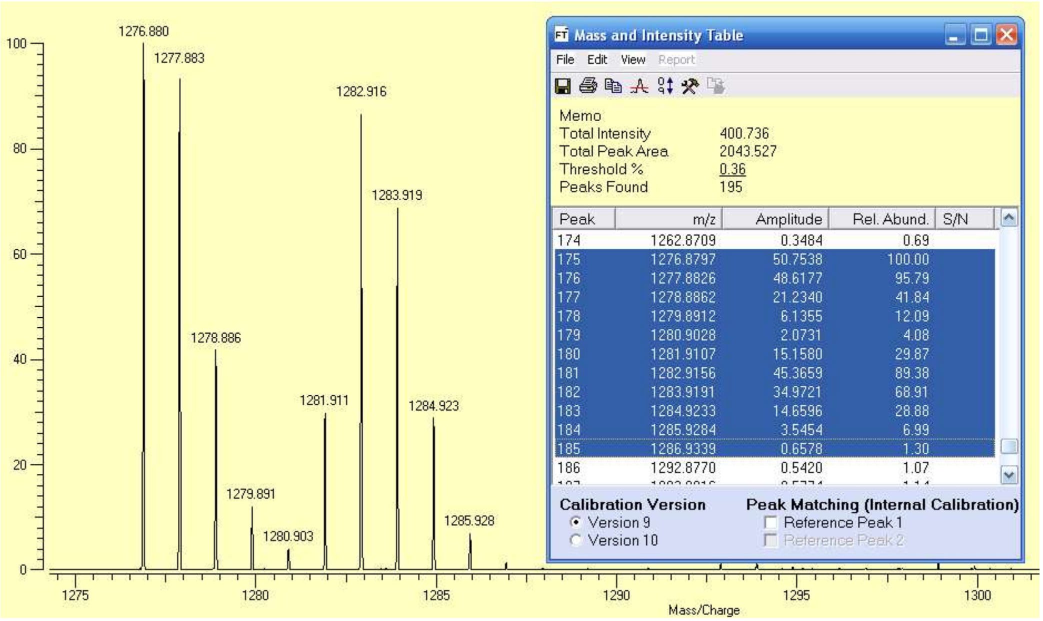

Peak	m/z	Amplitude	Rel. Abund.	S/N
174	1262.8709	0.3484	0.69	
175	1276.8797	50.7538	100.00	
176	1277.8826	48.6177	95.79	
177	1278.8862	21.2340	41.84	
178	1279.8912	6.1355	12.09	
179	1280.9028	2.0731	4.08	
180	1281.9107	15.1580	29.87	
181	1282.9156	45.3659	89.38	
182	1283.9191	34.9721	68.91	
183	1284.9233	14.6596	28.88	
184	1285.9284	3.5454	6.99	
185	1286.9339	0.6578	1.30	
186	1292.8770	0.5420	1.07	

General procedure for MS experiments (chiral recognition effect)

The chiral (enantiopure) calixarene derivative ($1.9 \cdot 10^{-3}$ mmol) were dissolved in 0.5 mL of $CHCl_3$ ($3.8 \cdot 10^{-3}$ M solution). Then, the barfate salt (*S*)-**10**$^{+} \cdot [B(Ar^F)_4]^-$ ($3.8 \cdot 10^{-3}$ mmol, $7.6 \cdot 10^{-3}$ M) and (*R*)-**10**-*d*$_6^{+} \cdot [B(Ar^F)_4]^-$ ($3.8 \cdot 10^{-3}$ mmol, $7.6 \cdot 10^{-3}$ M) were added and the mixture was stirred for 15 min. Then, the solution was diluted at the desired concentration with CH_2Cl_2 just before the MS analysis.

MS condition: sample concentration 300 μM; flow rate 2-4 μL/min; sample cone: 25 V; HV 2500 V; source temperature and temperature of desolvation gas were kept constant at 40 °C, no nebulizer gas was used for the experiments.

$I_S/I_{R\text{-}d_n}$ evaluation

The deuterated axle is a mixture of partially deuterated compounds so **I_{Sd_n}** can be evaluated as the sum of the intensity of each peak related to the deuterated compounds.

This operation is valid assuming that no significant differences and isotopoic effect occur between the partially deuterated compounds (d_4 and d_5) and the fully deuterated one (d_6). The intensity of the non-deuterated compounds, is than evaluated as the sum of each peak related to its distribution.

The corresponding peak intensities are pasted from the Omega software controlling the mass spectrometer to the spreadsheet, which calculates the intensity of each peak and the **$I_S/I_{R\text{-}d_n}$**.

Selected examples

1:2:2 mixture of (*R,R*)-**6**, (*S*)-**10**$^{+}$ ·[B(Ar^{F})$_4$]$^{-}$ and (*R*)-**10**-*d*$_6$$^{+}$·[B(Ar^{F})$_4$]$^{-}$

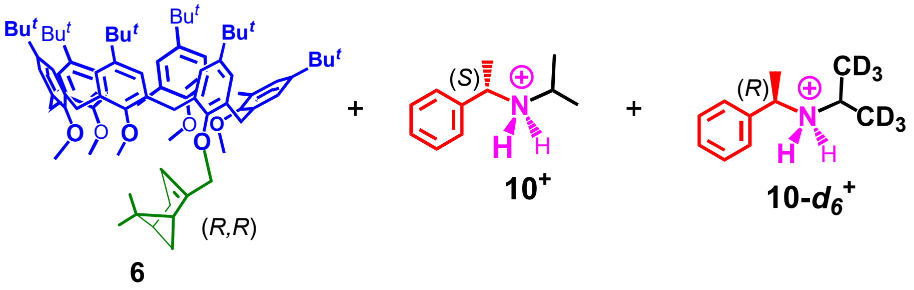

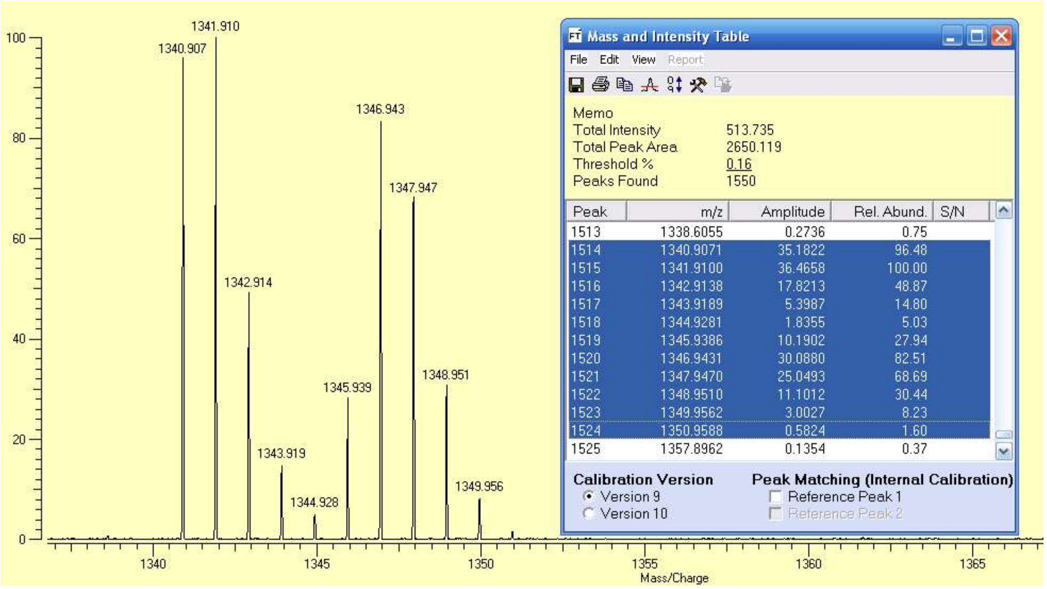

	Peak	*m/z*	Rel. Abund.
Non-Deuterated Complex	A	1340,9037	96,48
	A+1	1341,9070	100,00
	A+2	1342,9116	48,87
	A+3	1343,9166	14,80
	A+4	1344,9262	5,03
		I_R =	265,18

	Peak	*m/z*	Rel. Abund.
Deuterated Complex	A(D5)	1345,9369	27,94
	A (D5)+1 + A (D6)	1346,9401	82,51
	A (D5)+2 + A (D6)+1	1347,9440	68,69
	A (D5)+3 + A (D6)+2	1348,9493	30,44
	A (D5)+4 + A (D6)+3	1349,9548	8,23
	A (D6)+4	1350,9563	1,60
		I_{Rdn} =	219,41
		I_R/I_{Rdn} =	1,21

1:2:2 mixture of (*R*)-**7**, (*S*)-**10**$^+$ ·[B(Ar^F)$_4$]$^-$ and (*R*)-**10**-*d*$_6$$^+$·[B(Ar^F)$_4$]$^-$

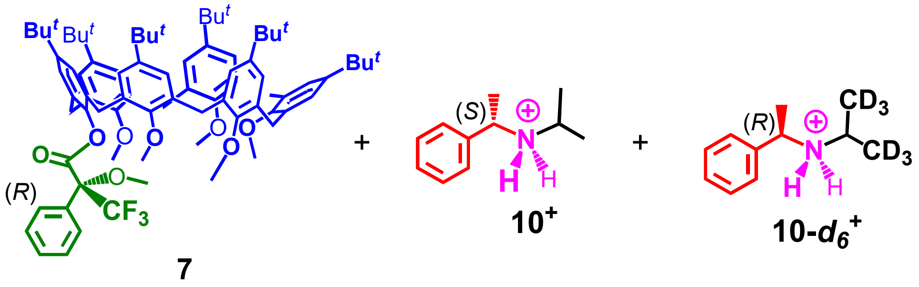

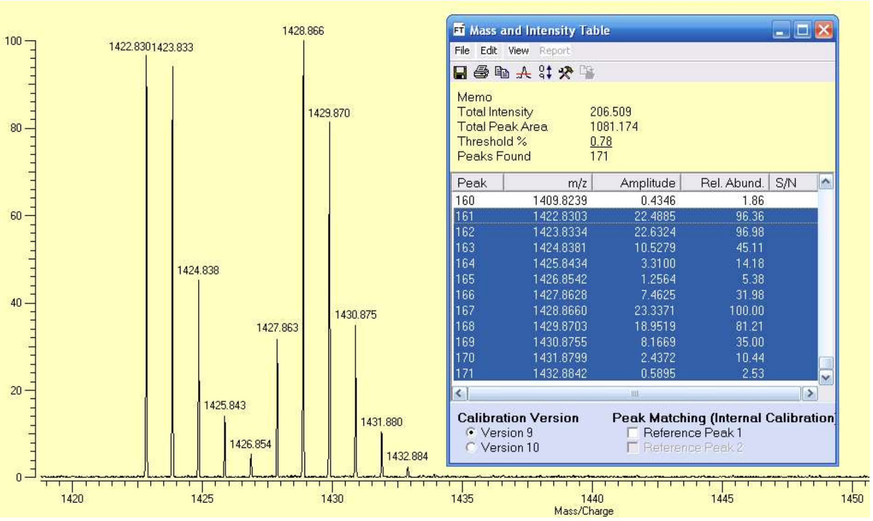

	Peak	*m/z*	Rel. Abund.
Non-Deuterated Complex	A	1340,9037	96,36
	A+1	1341,9070	96,98
	A+2	1342,9116	45,11
	A+3	1343,9166	14,18
	A+4	1344,9262	5,38
		I_R =	258,01

	Peak	*m/z*	Rel. Abund.
Deuterated Complex	A(D5)	1345,9369	31,98
	A (D5)+1 + A (D6)	1346,9401	100,00
	A (D5)+2 + A (D6)+1	1347,9440	81,21
	A (D5)+3 + A (D6)+2	1348,9493	35,00
	A (D5)+4 + A (D6)+3	1349,9548	10,44
	A (D6)+4	1350,9563	2,53
		I_{Rdn} =	261,16
		I_R/I_{Rdn} =	0,99

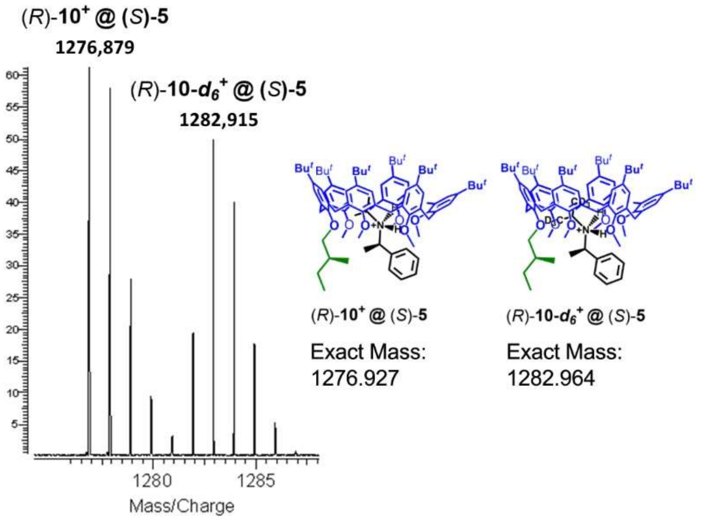

Figure S21. Significant portion of the mass spectrum of a 1:2:2 mixture (CH_2Cl_2, 300 μM) of derivatives (*S*)-**5**, (*R*)-$\mathbf{10^+}$·$[B(Ar^F)_4]^-$, and (*R*)-**10-*d*$_6$**$^{\mathbf{+}}$·$[B(Ar^F)_4]^-$, respectively.

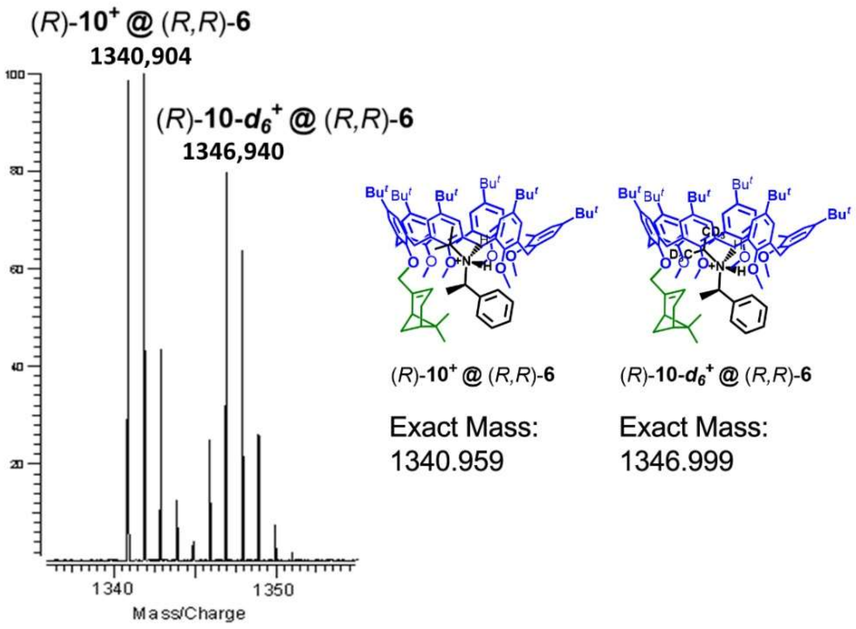

Figure S22. Significant portion of the mass spectrum of a 1:2:2 mixture (CH_2Cl_2, 300 μM) of derivatives (*R*)-**6**, (*R*)-$\mathbf{10^+}$·$[B(Ar^F)_4]^-$, and (*R*)-**10-*d*$_6$**$^{\mathbf{+}}$·$[B(Ar^F)_4]^-$, respectively.

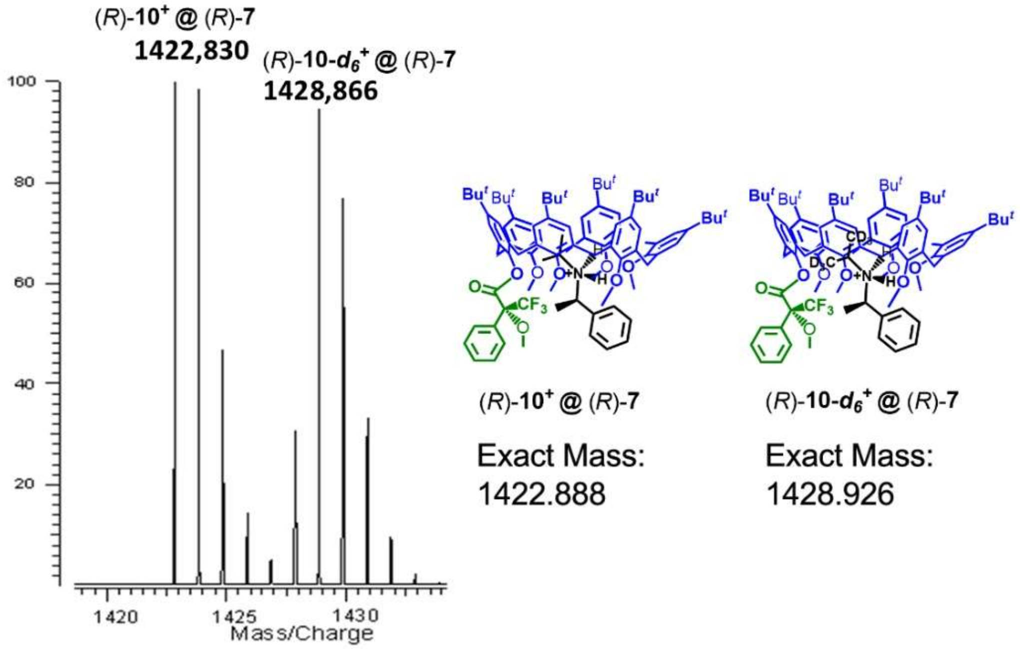

Figure S23. Significant portion of the mass spectrum of a 1:2:2 mixture (CH_2Cl_2, 300 μM) of derivatives (*R*)-**7**, (*R*)-$\mathbf{10^+}$·$[B(Ar^F)_4]^-$, and (*R*)-**10**-$\boldsymbol{d_6}^{\mathbf{+}}$·$[B(Ar^F)_4]^-$, respectively.

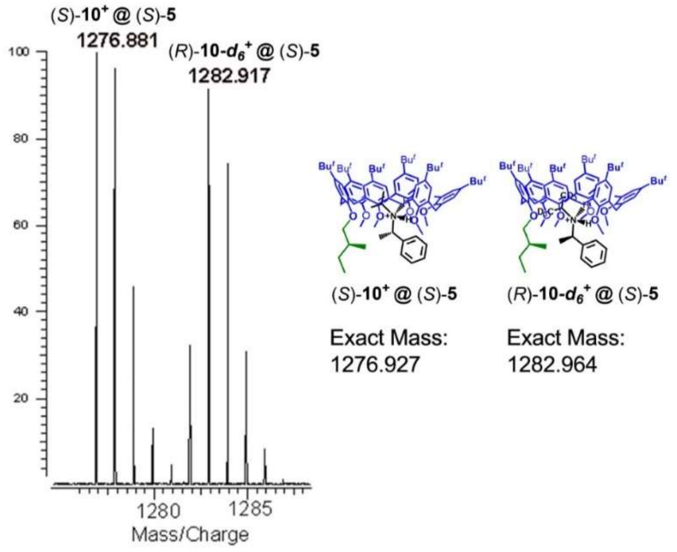

Figure S24. Significant portion of the mass spectrum of a 1:2:2 mixture (CH_2Cl_2, 300 μM) of derivatives (*S*)-**5**, (*S*)-$\mathbf{10^+}$·$[B(Ar^F)_4]^-$, and (*R*)-**10**-$\boldsymbol{d_6}^{\mathbf{+}}$·$[B(Ar^F)_4]^-$.

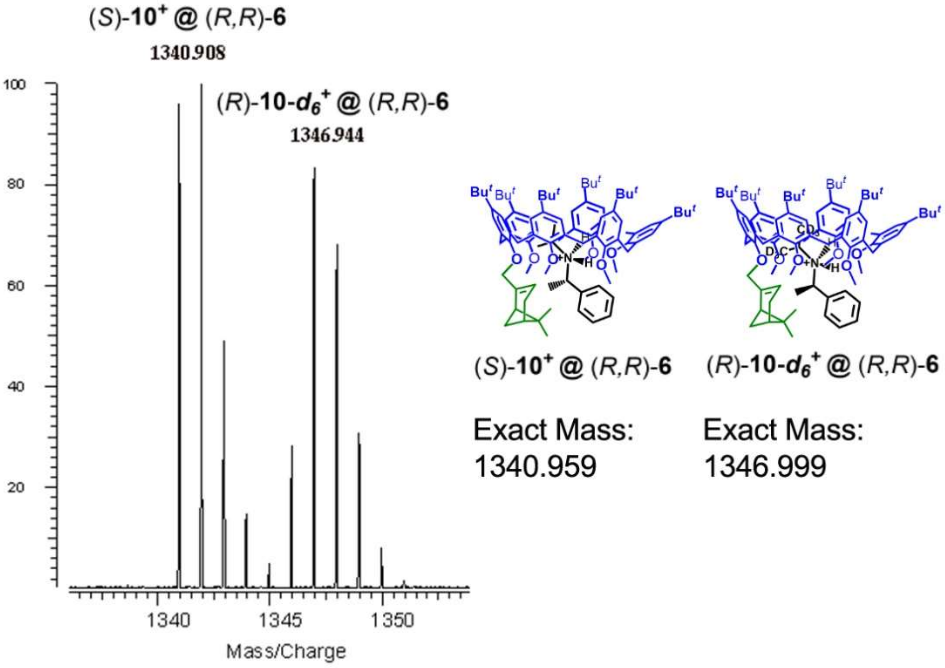

Figure S25. Significant portion of the mass spectrum of a 1:2:2 mixture (CH_2Cl_2, 300 μM) of derivatives (*R*)-**6**, (*S*)-$\mathbf{10^+}$·[B(Ar^F)$_4$]$^-$, and (*R*)-**10-**$\boldsymbol{d_6}^{\mathbf{+}}$·[B(Ar^F)$_4$]$^-$, respectively.

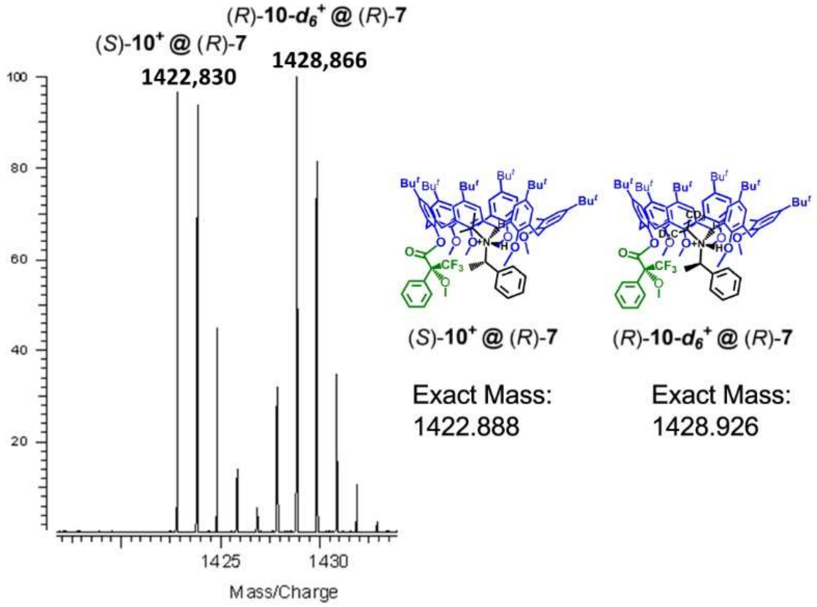

Figure S26. Significant portion of the mass spectrum of a 1:2:2 mixture (CH_2Cl_2, 300 μM) of derivatives (*R*)-**7**, (*S*)-$\mathbf{10^+}$·[B(Ar^F)$_4$]$^-$, and (*R*)-**10-**$\boldsymbol{d_6}^{\mathbf{+}}$·[B(Ar^F)$_4$]$^-$, respectively.

Cartesian Coordinates of the DFT-optimized structure of pseudorotaxanes at the B97D3/SVP/SVPFIT level of theory.

8$^+$@(*R*)-4 pseudo[2]rotaxane

```
O      -0.133000    1.200800   -3.367900
O       2.809000    3.707900   -0.929800
O       2.940000    1.203200    1.933700
C       5.209200    0.988100    1.141000
C       5.018800    2.225800    0.258700
H       4.294400    2.872500    0.757500
C       3.383500    2.673900   -1.657500
C       0.450300    3.524200   -3.047900
C       2.868600    2.403500   -2.939500
C       4.477400    0.558200   -3.170900
C       5.526600   -1.282800    2.745200
H       5.635300   -2.152400    3.387600
C      -1.857900    2.763700   -2.678300
C       6.613900   -0.852800    1.973300
C      -0.522200    2.509000   -3.029900
C       4.154600    0.524600    1.944500
C       4.291800   -0.627300    2.737600
C       4.409000    1.887200   -1.105300
C       1.856400    3.326200   -3.609200
H       1.766600    2.996000   -4.650000
H       2.303300    4.324100   -3.672600
C       3.429900    1.351000   -3.668000
H       3.062300    1.179200   -4.676000
C       6.418700    0.278000    1.172200
H       7.240100    0.638600    0.567600
C       0.046700    4.809400   -2.680300
H       0.788500    5.601300   -2.719100
C       5.140900   -0.506500   -4.067500
C      -2.198900    4.076500   -2.320900
H      -3.228200    4.283200   -2.062600
C       6.318800    3.053200    0.068100
H       7.093300    2.434900   -0.401700
H       6.113500    3.844900   -0.661700
C       4.928000    0.838300   -1.877700
H       5.720900    0.243100   -1.442700
C      -1.271700    5.121900   -2.316200
C      -2.930200    1.686300   -2.857800
H      -3.575000    2.020400   -3.681900
H      -2.432100    0.783800   -3.200400
C       7.957100   -1.604100    2.047500
```

C	-1.659400	6.584000	-2.013900
C	6.860500	3.686000	1.355900
H	6.083900	4.329000	1.796600
H	7.061600	2.903900	2.098300
C	2.821800	2.210300	2.947800
H	2.892300	1.776000	3.951500
H	1.841100	2.669100	2.817200
H	3.601200	2.973900	2.842400
C	-0.267500	0.872900	-4.760400
H	0.424100	1.460100	-5.371600
H	-1.289400	1.049900	-5.109300
H	-0.031300	-0.187500	-4.859400
C	3.434200	4.992100	-1.020100
H	4.326600	5.050000	-0.388800
H	2.701000	5.720100	-0.666600
H	3.715200	5.232000	-2.051600
C	6.229700	-1.303900	-3.324400
H	7.049000	-0.661000	-2.988300
H	6.658700	-2.054700	-3.995100
H	5.828200	-1.829900	-2.451600
C	-1.375100	7.444900	-3.269200
H	-0.315400	7.438100	-3.541400
H	-1.663600	8.485400	-3.087000
H	-1.944300	7.081400	-4.130800
C	8.496800	-1.539900	3.496600
H	8.661500	-0.503500	3.808200
H	9.451000	-2.072400	3.571100
H	7.804900	-1.997300	4.209800
C	8.133000	4.513600	1.132800
H	8.907100	3.872300	0.689900
H	7.931600	5.298700	0.391200
C	9.021400	-1.000100	1.111800
H	8.712200	-1.037100	0.061700
H	9.952200	-1.568500	1.199300
H	9.248500	0.039900	1.367200
C	7.749900	-3.083500	1.645800
H	7.042500	-3.591500	2.308200
H	8.698800	-3.627800	1.698400
H	7.370600	-3.163500	0.621600
C	4.089300	-1.511800	-4.587000
H	3.633500	-2.065000	-3.758500
H	4.557300	-2.243200	-5.254200
H	3.293500	-1.016300	-5.152900
C	5.800100	0.203100	-5.275700
H	5.065800	0.745800	-5.878500
H	6.292700	-0.527900	-5.925900
H	6.554400	0.922400	-4.942200
C	-0.828600	7.127600	-0.827700
H	-1.082700	6.612200	0.103500
H	-1.037000	8.192600	-0.680100
H	0.247700	7.023800	-0.997000
C	-3.150100	6.734300	-1.656300

H	-3.801200	6.424000	-2.480200
H	-3.370900	7.785200	-1.446100
H	-3.414700	6.158100	-0.765400
C	8.670500	5.149800	2.417800
H	7.931900	5.823500	2.866800
H	9.576500	5.732400	2.225200
H	8.918300	4.387200	3.164600
O	0.511500	-1.700900	4.467100
O	-2.441400	-3.655000	1.037200
O	-2.847400	-0.891900	-1.627100
C	-4.695700	-0.397300	-0.135100
C	-4.622800	-1.807500	0.467100
H	-4.029600	-2.416400	-0.216900
C	-2.862800	-2.790800	2.048500
C	0.270200	-3.616000	3.031700
C	-2.214600	-2.862400	3.296200
C	-3.675800	-1.077800	4.137300
C	-4.853700	2.173900	-1.256600
H	-4.934100	3.145000	-1.727600
C	2.346000	-2.316800	3.021100
C	-5.784600	1.809000	-0.280500
C	1.049000	-2.548500	3.510700
C	-3.798500	0.017600	-1.137900
C	-3.847300	1.304100	-1.699000
C	-3.889400	-1.858700	1.814600
C	-1.125000	-3.889400	3.580100
H	-1.058800	-3.996600	4.667900
H	-1.438500	-4.867200	3.203700
C	-2.637800	-2.003700	4.310500
H	-2.139300	-2.074100	5.270700
C	-5.672100	0.521500	0.265000
H	-6.402800	0.209900	1.002600
C	0.848200	-4.491600	2.106900
H	0.258200	-5.343500	1.783400
C	-4.110400	-0.184800	5.315700
C	2.873600	-3.214800	2.084600
H	3.880100	-3.036900	1.729200
C	-6.019700	-2.474700	0.601400
H	-6.652500	-1.877100	1.268600
H	-5.888900	-3.432900	1.118800
C	-4.275100	-1.024500	2.876100
H	-5.074000	-0.316700	2.697800
C	2.162600	-4.337500	1.637800
C	3.151400	-1.148000	3.588600
H	3.575000	-1.464000	4.551200
H	2.446500	-0.348400	3.818100
C	-6.942500	2.727800	0.157700
C	2.823200	-5.436000	0.778100
C	-6.748700	-2.715300	-0.725600
H	-6.129100	-3.358000	-1.369300
H	-6.866200	-1.765800	-1.263100
C	-3.338800	-1.645300	-2.752800

H	-2.496900	-2.199000	-3.164500
H	-4.120100	-2.345300	-2.441000
H	-3.750200	-0.976800	-3.515500
C	0.859100	-2.022300	5.817500
H	0.322200	-1.314500	6.452200
H	0.558600	-3.044400	6.078400
H	1.934500	-1.917800	5.998900
C	-3.161500	-4.890800	0.963100
H	-3.267000	-5.353800	1.950300
H	-4.158900	-4.750800	0.532200
H	-2.583000	-5.556200	0.317600
C	-5.308700	0.716600	4.962700
H	-6.183500	0.132700	4.657700
H	-5.596400	1.304300	5.839700
H	-5.070800	1.426700	4.163900
C	3.116800	-6.646700	1.699100
H	2.196800	-7.048200	2.134700
H	3.601600	-7.450000	1.133200
H	3.779700	-6.362100	2.522200
C	-6.870400	4.117000	-0.504100
H	-6.950500	4.057100	-1.594500
H	-7.703200	4.733100	-0.152000
H	-5.943300	4.638700	-0.249100
C	-8.126000	-3.368600	-0.547000
H	-8.748600	-2.726100	0.089900
H	-8.012000	-4.315200	-0.001700
C	-8.284800	2.069600	-0.244000
H	-8.421500	1.094600	0.233600
H	-9.123600	2.706700	0.056000
H	-8.342300	1.922500	-1.327400
C	-6.910300	2.926700	1.691500
H	-5.980700	3.407700	2.007100
H	-7.743400	3.564800	2.004700
H	-7.005000	1.979900	2.231400
C	-2.931300	0.724300	5.737300
H	-2.650800	1.406400	4.928500
H	-3.210800	1.329700	6.606400
H	-2.044700	0.141900	6.004900
C	-4.521800	-1.079100	6.510000
H	-3.696200	-1.707200	6.857000
H	-4.842900	-0.459600	7.354000
H	-5.352300	-1.738900	6.238900
C	1.899000	-5.898400	-0.368500
H	1.693800	-5.084600	-1.070000
H	2.377200	-6.708600	-0.928700
H	0.940400	-6.279900	-0.006100
C	4.151800	-4.969600	0.150600
H	4.908500	-4.738000	0.905500
H	4.559000	-5.764700	-0.481700
H	4.015800	-4.081600	-0.476900
C	-8.846800	-3.625700	-1.873300
H	-8.267700	-4.297600	-2.516900

H	-9.826500	-4.085400	-1.711400
H	-9.004700	-2.692900	-2.426200
C	-1.930600	3.242800	1.672800
C	-2.324500	1.911700	1.822000
C	-1.509000	0.892700	1.342100
C	-0.311800	1.194800	0.682400
C	0.079900	2.530800	0.540800
C	-0.724300	3.551600	1.042600
H	-3.255400	1.666200	2.316700
H	-1.807400	-0.138800	1.500500
H	1.018500	2.784300	0.060900
H	-0.409500	4.582800	0.943200
C	0.575400	0.084600	0.188700
H	1.576500	0.448200	-0.042600
H	0.664300	-0.706100	0.937200
N	0.051300	-0.596300	-1.070500
H	0.029800	0.073100	-1.867400
H	-0.937100	-0.886500	-0.959900
C	0.921900	-1.783300	-1.442500
H	1.831400	-1.364800	-1.878900
H	1.186500	-2.274000	-0.502700
C	-0.962000	-4.633700	-4.063400
C	-0.067500	-3.699800	-4.588300
C	0.535700	-2.770200	-3.741500
C	0.245800	-2.759600	-2.371700
C	-0.647800	-3.708400	-1.851900
C	-1.247700	-4.640100	-2.695300
H	0.167800	-3.712600	-5.646600
H	1.256700	-2.067100	-4.145700
H	-0.878300	-3.717300	-0.789800
H	-1.931100	-5.380500	-2.294200
C	-1.674900	-5.599500	-4.977300
F	-0.972100	-5.839200	-6.101300
F	-1.903400	-6.780800	-4.368400
F	-2.877600	-5.105100	-5.351700
C	-2.836100	4.341400	2.155200
F	-3.767800	4.666800	1.216000
F	-3.522300	3.981300	3.262500
F	-2.161200	5.474900	2.437600

Endo-chiral (*S*)-**9⁺@**(*R*)-**4**

O	-2.87580	3.14560	3.23430
O	1.06060	2.02630	2.97200
O	-4.09230	-0.30890	1.49490
O	-2.58900	-3.79850	0.57299
O	1.76720	-3.57870	1.24200
O	3.03630	-0.93390	2.16529
C	1.70170	3.08600	2.32629
C	1.83960	-4.19940	-2.39909
H	1.15270	-4.55710	-3.16960
C	3.05730	2.95950	1.96120
C	-1.36100	4.40620	0.11720
H	-0.43660	4.89600	-0.20130
C	1.34540	-4.08790	-1.08490
C	3.64600	4.00110	1.22210
H	4.69280	3.89670	0.93470
C	2.22929	-3.68880	-0.06190
C	0.97790	4.26420	2.04490
C	-2.35639	-3.19270	-0.64290
C	-5.46220	1.38660	0.44200
C	1.61810	5.27930	1.31730
H	1.05519	6.19790	1.11910
C	4.52620	0.83270	1.45820
C	-3.70930	3.12370	0.96650
C	-0.87250	-2.57260	-2.45070
H	0.09510	-2.66760	-2.94550
C	3.88310	1.78370	2.45410
H	4.70160	2.20290	3.07080
H	3.25550	1.19029	3.12490
C	-1.51300	4.09410	1.47650
C	-2.70710	3.46360	1.89530
C	-2.35429	4.11250	-0.83730
C	4.15590	-0.52590	1.43830
C	-3.50820	3.45200	-0.38660
H	-4.28580	3.16870	-1.09759
C	-1.11460	-3.35260	-1.31010
C	-6.32060	1.75130	-0.61370
H	-6.69350	2.77930	-0.62870
C	3.57690	-3.38680	-0.35600
C	-1.80770	-1.64960	-2.95760
C	-0.10690	-4.37230	-0.78660
H	-0.25210	-4.46790	0.29700
C	2.94410	5.16070	0.85880
C	-4.96800	0.07110	0.48580
C	-3.33810	-2.31710	-1.15280
C	4.88720	-1.47470	0.69470
C	4.00800	-3.46300	-1.68860
H	5.04940	-3.20410	-1.90580
C	-0.45030	4.44470	2.50710
H	-0.64610	3.84240	3.40390

C	-5.31990	-0.86480	-0.50839
C	4.54730	-2.95510	0.72810
H	4.13480	-3.22550	1.70710
H	5.48550	-3.52270	0.60180
C	-3.03930	-1.55000	-2.29270
H	-3.80300	-0.85540	-2.64870
C	3.58010	6.29530	0.03400
C	-6.16780	-0.45470	-1.54790
H	-6.42020	-1.18950	-2.32090
C	-3.53420	4.14799	4.00699
H	-3.60150	3.76810	5.03979
H	-2.96490	5.09820	4.00520
C	5.95010	-1.01670	-0.09670
H	6.52380	-1.75780	-0.66340
C	3.16190	-3.86870	-2.73750
C	6.30560	0.34330	-0.17400
C	-6.68060	0.85540	-1.63370
C	5.59070	1.24110	0.63400
H	5.88230	2.29199	0.65490
C	1.82960	-4.79120	1.99910
H	1.41050	-4.57390	2.99360
H	2.87450	-5.14250	2.10400
C	-2.15870	4.53300	-2.30670
C	7.47310	0.78340	-1.07710
C	3.69520	-3.92760	-4.18079
C	-0.86690	3.89520	-2.86870
H	0.02800	4.22660	-2.31670
H	-0.72830	4.17800	-3.92760
H	-0.90590	2.79480	-2.81280
C	-2.03510	6.07520	-2.36930
H	-1.90350	6.40890	-3.41460
H	-1.16850	6.43700	-1.78900
C	4.19600	-2.52080	-4.59000
H	4.57139	-2.53450	-5.62870
H	5.02030	-2.17380	-3.94330
H	3.37990	-1.77950	-4.52790
C	1.14380	2.02530	4.40409
H	2.17970	2.21790	4.73710
H	0.47870	2.79230	4.84199
C	4.95620	5.89440	-0.53340
H	5.69240	5.70090	0.26600
H	5.35670	6.71260	-1.15609
H	4.88910	4.99120	-1.16570
C	2.65560	6.65930	-1.15370
H	2.49630	5.79120	-1.81790
H	3.10760	7.46740	-1.75550
H	1.66580	7.01220	-0.81750
C	-3.34410	4.10290	-3.19260
H	-3.16240	4.41099	-4.23660
H	-4.28950	4.57070	-2.86660
H	-3.48450	3.00870	-3.18740
C	-1.47440	-0.83780	-4.22400

C	3.75890	7.53410	0.94480
H	2.79190	7.88340	1.34650
H	4.21279	8.36800	0.37950
H	4.41600	7.29860	1.80050
C	4.86489	-4.93800	-4.25330
H	4.52960	-5.94800	-3.95850
H	5.69620	-4.64930	-3.58720
H	5.26260	-4.99320	-5.28250
C	2.60920	-4.36190	-5.18350
H	2.22860	-5.37470	-4.96400
H	3.02940	-4.38000	-6.20380
H	1.75390	-3.66290	-5.18919
C	8.78640	0.17430	-0.52859
H	8.74949	-0.92880	-0.51880
H	8.97439	0.51590	0.50449
H	9.64460	0.47840	-1.15480
C	7.23500	0.27789	-2.52080
H	7.14920	-0.82110	-2.56619
H	8.07740	0.57170	-3.17190
H	6.31130	0.70740	-2.94510
C	-2.52430	0.25720	-4.49280
H	-2.22880	0.85620	-5.37130
H	-2.61870	0.94410	-3.63440
H	-3.52200	-0.16560	-4.70059
C	-0.09110	-0.16070	-4.08200
H	0.72130	-0.89350	-3.94470
H	-0.06200	0.52470	-3.21920
H	0.14080	0.42460	-4.98940
C	7.62330	2.31650	-1.12360
H	8.43960	2.59140	-1.81340
H	7.87470	2.73610	-0.13400
H	6.70050	2.80610	-1.48160
C	-1.44470	-1.80770	-5.43080
H	-0.67630	-2.58930	-5.29700
H	-1.21520	-1.26020	-6.36310
H	-2.42010	-2.31029	-5.55550
H	-0.36040	-5.36090	-1.21790
C	-7.55140	1.26180	-2.83650
C	-6.71310	1.09740	-4.12899
C	-8.80059	0.35190	-2.90480
C	-8.01880	2.72690	-2.74780
H	-8.64010	2.97400	-3.62600
H	-8.62940	2.90790	-1.84590
H	-7.16620	3.42900	-2.73710
H	-7.30510	1.39440	-5.01340
H	-5.80820	1.73010	-4.09159
H	-6.38940	0.05270	-4.27710
H	-9.43220	0.63030	-3.76750
H	-8.52870	-0.71150	-3.01860
H	-9.40750	0.45030	-1.98740
H	-0.58320	5.50340	2.80260
H	0.82080	1.03130	4.74600

H	-4.55470	4.34750	3.62640
H	1.22820	-5.59350	1.53420
C	3.27020	-1.41490	3.49020
H	2.28290	-1.57550	3.94930
H	3.83120	-2.36720	3.48420
H	3.83460	-0.67470	4.08720
C	-4.72790	-0.88490	2.63720
H	-5.35130	-1.75540	2.35619
H	-3.92940	-1.21080	3.32130
H	-5.37160	-0.14530	3.15260
C	-4.71340	-2.24650	-0.50320
H	-5.38760	-2.93020	-1.05360
H	-4.63220	-2.64210	0.51720
C	-4.98400	2.42600	1.42850
H	-5.77850	3.18440	1.56450
H	-4.79350	1.97980	2.41500
C	2.22260	-0.28699	-1.32050
C	3.33120	0.25520	-1.97800
C	3.47500	1.64650	-2.07460
C	2.51180	2.48749	-1.50270
C	1.40800	1.94480	-0.83220
C	1.25040	0.55120	-0.74240
H	4.07810	-0.41230	-2.41420
H	4.33779	2.07620	-2.59520
H	2.61790	3.57300	-1.57080
H	0.66590	2.61510	-0.39370
C	-0.64310	-1.53070	5.84890
C	-0.37290	-1.84160	4.50699
C	-0.86650	-1.02550	3.47300
C	-1.63790	0.10780	3.79900
C	-1.90660	0.41880	5.13730
C	-1.40970	-0.39840	6.16560
H	-0.25950	-2.17640	6.64730
H	0.20470	-2.73780	4.25530
H	-2.04460	0.74590	3.00870
H	-2.50760	1.30410	5.36840
H	-1.62300	-0.15690	7.21320
C	-0.57970	-6.72920	4.08920
C	-0.65440	-7.49160	2.91380
C	-1.34800	-6.99730	1.79860
C	-1.97240	-5.73840	1.84290
C	-1.88790	-4.97560	3.02350
C	-1.19870	-5.47000	4.13970
H	-0.04370	-7.11670	4.96330
H	-0.17130	-8.47430	2.86380
H	-1.40260	-7.59690	0.88110
H	-2.36180	-3.98930	3.06170
H	-1.15180	-4.87059	5.05660
N	0.35190	-0.35060	1.39580
H	1.33390	-0.69420	1.50770
H	0.36060	0.54940	1.92420
C	-2.76820	-5.23130	0.65390

C	-1.24330	0.72950	-0.18160
H	-2.10410	0.19310	0.23990
H	-1.15950	1.70240	0.32560
H	-1.46300	0.91560	-1.24170
C	-0.59780	-1.34760	2.02789
H	-0.14580	-2.33880	1.88920
H	-1.53610	-1.31180	1.45740
C	0.04450	-0.07720	-0.07620
H	-0.10860	-1.07749	-0.51070
H	-2.94120	6.55880	-1.96380
H	2.10170	-1.37220	-1.26870
C	-4.25770	-5.56330	0.77570
H	-2.37800	-5.69680	-0.26889
H	-4.39590	-6.65350	0.87860
H	-4.68499	-5.07599	1.66890
H	-4.80809	-5.22320	-0.11750

REFERENCES

[1] (a) Gaeta, C.; Troisi, F.; Neri, P. *endo-Cavity* Complexation and Through-the-Annulus Threading of Large Calixarenes Induced by Very Loose Alkylammonium Ion Pairs. *Org. Lett.* **2010**, *12*, 2092–2095. (b) Gaeta, C.; Talotta, C.; Neri, P. Calix[6]arene-based atropoisomeric pseudo[2]rotaxanes. *Beilstein J. Org. Chem* **2018**, *14,* 2112–2124. (c) Gaeta, C.; Talotta, C.; Neri, P. Pseudorotaxane orientational stereoisomerism driven by π-electron density. *Chem. Commun*. **2014**, *50*, 9917-9920.
